# Peptidoglycan hydrolysis mediated by the amidase AmiC and its LytM activator NlpD is critical for cell separation and virulence in the phytopathogen *Xanthomonas campestris*

**DOI:** 10.1111/mpp.12653

**Published:** 2018-02-01

**Authors:** Li‐Chao Yang, Yong‐Liang Gan, Li‐Yan Yang, Bo‐Le Jiang, Ji‐Liang Tang

**Affiliations:** ^1^ State Key Laboratory for Conservation and Utilization of Subtropical Agro‐bioresources, College of Life Science and Technology Guangxi University Nanning Guangxi 530004 China

**Keywords:** amidase, cell separation, LytM protein, peptidoglycan hydrolysis, T3SS, virulence, *Xanthomonas*

## Abstract

The essential stages of bacterial cell separation are described as the synthesis and hydrolysis of septal peptidoglycan (PG). The amidase, AmiC, which cleaves the peptide side‐chains linked to the glycan strands, contributes critically to this process and has been studied extensively in model strains of *Escherichia coli*. However, insights into the contribution of this protein to other processes in the bacterial cell have been limited. *Xanthomonas campestris* pv. *campestris* (*Xcc*) is a phytopathogen that causes black rot disease in many economically important plants. We investigated how AmiC and LytM family regulators, NlpD and EnvC, contribute to virulence and cell separation in this organism. Biochemical analyses of purified AmiC demonstrated that it could hydrolyse PG and its activity could be potentiated by the presence of the regulator NlpD. We also established that deletion of the genes encoding *amiC1* or *nlpD* led to a reduction in virulence as well as effects on colony‐forming units and cell morphology. Moreover, further genetic and biochemical evidence showed that AmiC1 and NlpD affect the secretion of type III effector XC3176 and hypersensitive response (HR) induction *in planta*. These findings indicate that, in addition to their well‐studied role(s) in cell separation, AmiC and NlpD make an important contribution to the type III secretion (T3S) and virulence regulation in this important plant pathogen.

## Introduction

Gram‐negative pathogenic bacteria have evolved various secretion systems, such as the type II (T2S), type III (T3S) and type IV (T4S) secretion systems, which span both the bacterial inner and outer membranes, to transfer virulence factors into host cells and cause disease (Costa *et al*., 2015). In the periplasmic space between the outer and inner membranes, there is a tight peptidoglycan (PG) layer, which is essential for the preservation of cell integrity by withstanding turgor and maintaining a defined cell shape (Silhavy *et al*., 2010; Vollmer *et al*., 2008). However, the PG layer can also be a barrier impeding the assembly of the membrane‐spanning apparatuses of the secretion systems. Therefore, the PG layer needs to be locally remodelled by PG‐lytic enzymes to provide space and acceptor sites for the secretion apparatus assembly (Burkinshaw and Strynadka, 2014; Koraimann, 2003; Scheurwater and Burrows, 2011). A typical Gram‐negative bacterial PG layer is composed of glycan strands of β‐1,4‐glycosidic bond‐linked *N*‐acetylglucosamine (GlcNAc) and *N*‐acetylmuramic acid (MurNAc) disaccharide, which are cross‐linked by short peptides to create the meshwork layer (Silhavy *et al*., 2010; Vollmer *et al*., 2008). Bacteria possess two main classes of PG‐lytic enzymes: the glycosidases that cleave the glycan backbone and the amidases (or peptidases) that cleave the peptide side‐chain (Firczuk and Bochtler, 2007; Frirdich and Gaynor, 2013; Vollmer *et al*., 2008). Previous studies have revealed that the lytic transglycosylase EtgA of *Escherichia coli* is required for efficient T3S. Purified recombinant EtgA can degrade PG *in vitro* and the mutant strain lacking EtgA is attenuated for T3S, suggesting that the lytic transglycosylase EtgA may contribute to PG rearrangement by selectively cleaving the backbone (Burkinshaw *et al*., 2015; García‐Gómez *et al*., 2011).

It is not yet clear whether the PG amidases are required for the assembly of bacterial secretion systems, such as the T3S system (T3SS), although their significance in cell division has been well established. Previous studies have revealed that the *E. coli N*‐acetylmuramoyl‐l‐alanine amidases AmiA, AmiB and AmiC are indispensable for daughter cell separation and their PG hydrolytic activity is activated by the LytM factors NlpD or EnvC. Inactivation of the genes encoding the amidases and the LytM factors results in a defect in cell separation, leading to the formation of long cell chains (Heidrich *et al*., 2001; Uehara *et al*., 2009, 2010). NlpD and EnvC are members of the LytM family proteins, which contain a LytM (lysostaphin/peptidase M23) domain and are widely distributed in bacteria (Firczuk and Bochtler, 2007). Although *E. coli* NlpD and EnvC do not seem to have any PG degradation activity themselves (Peters *et al*., 2013; Uehara *et al*., 2009), LytM members with PG hydrolytic activity have been found in other bacteria (Collier, 2010; Möll *et al*., 2010; Odintsov *et al*., 2004; Sycuro *et al*., 2010).

Nevertheless, despite significant progress, insights into the contribution of LytM family regulators and the amidase enzymes they control, and their link to virulence, remain incompletely understood. To this end, we examined how AmiC and its LytM family regulators contribute to virulence, T3S and cell separation in the important phytopathogen *Xanthomonas campestris* pv. *campestris* (*Xcc*). The pathogenicity of *Xcc* is dependent on the T3SS as the pathogen translocates effector proteins into host cells to interfere with host cell functions (He *et al*., 2007; Mansfield *et al*., 2012; Ryan *et al*., 2011). The *Xcc* T3S apparatus is composed of numerous proteins encoded by a cluster of *hrp* genes, which consists of at least six operons (*hrpA* to *hrpF*) (Arlat *et al*., 1991; Huang *et al*., 2009).

The aim of this work was to investigate whether these putative LytM factors and amidases are involved in *Xcc* pathogenicity. Here, we report the finding that one of the LytM proteins and one of the amidases are essential for *Xcc* virulence, homologous to the *E. coli* NlpD and AmiC, respectively. Furthermore, we provide evidence showing that the *Xcc* AmiC1 coupled with its activator NlpD contributes to T3S, in addition to cell division processes.

## Results

### *Xcc* encodes an NlpD‐like protein that is essential for *Xcc* virulence

A SMART (Simple Modular Architecture Research Tool) analysis (http://smart.embl-heidelberg.de) revealed that 10 open reading frames (ORFs) in the genome of *Xcc* strain 8004 (Qian *et al*., 2005) encode proteins with an identifiable LytM domain (i.e. peptidase_M23 domain) (Table S1, see Supporting Information). These ORFs are highly conserved in other fully sequenced genomes of *Xcc* strains. Amongst the 10 putative LytM proteins of *Xcc*, only XC2522 has a LysM domain and was named as NlpD based on its 59% amino acid identity with the *E. coli* NlpD (Table S1). We named XC0022 and XC3926 as EnvC and YebA based on their homology with *E. coli* EnvC and YebA proteins, respectively (Table S1).

To evaluate whether any of the predicted LytM proteins in *Xcc* strain 8004 are required for virulence, we employed a reverse genetics approach. A defined genome‐wide mutant library derived from the *Xcc* wild‐type strain 8004 has been generated previously by transposon (Tn5gusA5) insertion and homologous suicide plasmid (pK18mob) integration mutagenesis in our laboratory (Qian *et al*., 2005). One mutant strain for each of the genes encoding the predicted LytM proteins was obtained (Table S2, see Supporting Information). The virulence of each mutant strain was determined in the host plant Chinese radish by the leaf clipping inoculation method. Six days after inoculation, nine of the mutant strains caused visible disease symptoms, similar to the wild‐type strain 8004; however, the *nlpD* mutant (NK2522) did not cause any black rot disease symptoms, even at 10 days post‐inoculation. The lesion lengths induced by different strains were measured and are presented in Fig. S1 (see Supporting Information). As a result of its importance, the transcriptional start site of the *nlpD* gene was further evaluated by 5′ rapid amplification of cDNA ends (5′ RACE) analysis (Fig. S2, see Supporting Information). Taken together, these results suggest that NlpD is required for the virulence of *Xcc*.

**Figure 1:**
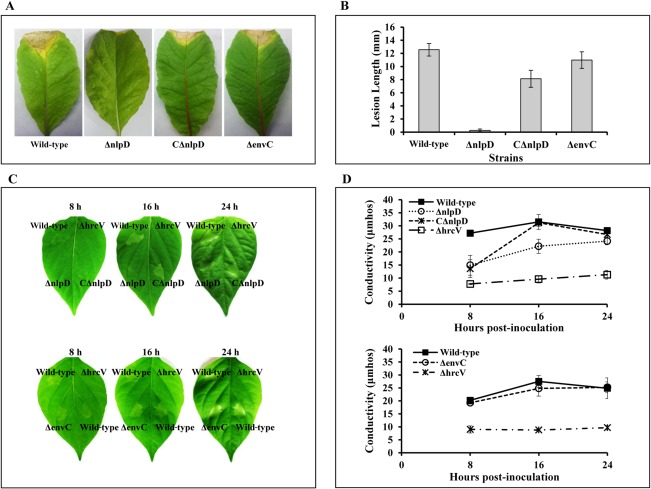
NlpD is required for pathogenicity and hypersensitive response (HR) induction of *Xanthomonas campestris* pv. *campestris* (*Xcc*). *Xcc* cells cultured overnight were washed and resuspended in double‐distilled H_2_O to an optical density at 600 nm (OD_600_) of 0.001 for the virulence test and 0.01 for the HR test. (A) Disease symptoms on Chinese radish (*Raphanus sativus*) leaves inoculated by cutting leaves with scissors dipped in the bacterial suspensions. (B) Lesion lengths were scored at 10 days post‐inoculation. Values represent the means and standard deviations from 60 inoculated leaves in one experiment. The experiment was repeated three times with similar results. (C) HR symptoms induced in pepper leaves (*Capsicum annuum* cv. ECW‐10R). Bacterial resuspension was infiltrated into the leaf mesophyll tissue with a blunt‐end plastic syringe. Photographs were taken at 8, 16 and 24 h after infiltration. Three plants were used in each experiment, and the experiment was repeated three times. The results presented are from a representative experiment, and similar results were obtained in all other independent experiments. The *hrcV* deletion mutant ΔhrcV was used as a negative control. (D) Electrolyte leakage from pepper leaves inoculated with *Xcc* strains. For each sample, three leaf discs were removed with a 0.7‐cm‐diameter cork borer from the infiltrated area. Three samples were taken for each measurement in each experiment. Results presented are from a representative experiment, and similar results were obtained in other experiments.

To facilitate further studies, a *nlpD* deletion mutant, named ΔnlpD (Table S2), was constructed by double‐crossover homologous recombination using the suicide plasmid pk18mobsacB (Table S2). Simultaneously, a complemented strain, named CΔnlpD (Table S2), was created by *in trans* expression of the *nlpD* gene cloned into the vector pLAFR6. Like the mutant strain NK2522 from the library, ΔnlpD showed highly reduced virulence to the host plant Chinese radish (Fig. 1A,B). Notably, complementation partly restored virulence to the ΔnlpD strain as shown in Fig. 1A,B. The ΔnlpD and CΔnlpD strains displayed a similar growth rate in the nutrient‐rich medium NYG, but a reduced growth rate in the minimal medium MMX, compared with the wild‐type strain (Fig. S3, see Supporting Information). The *envC* deletion mutant strain ΔenvC (Table S2) induced virulence symptoms similar to those of the wild‐type (Fig. 1A,B).

### NlpD and EnvC are required for *Xcc* cell morphology and separation

**Figure 2:**
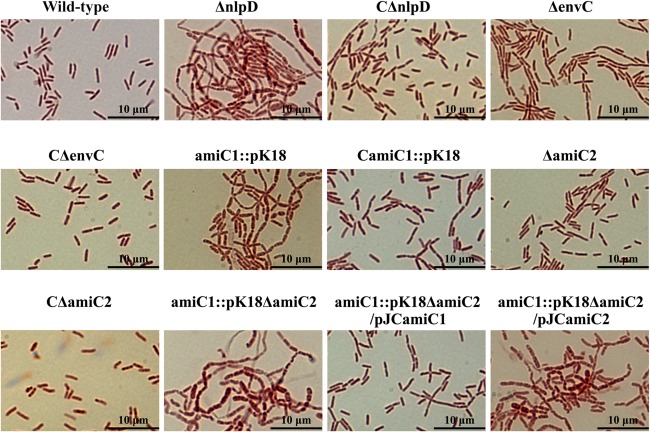
*Xanthomonas campestris* pv. *campestris* (*Xcc*) NlpD and AmiC1 are important for cell separation. Bacterial cells of *Xcc* strains cultured overnight in NYG medium were collected by centrifugation at 13 800 *g* for 30 s and resuspended in double‐distilled H_2_O. A 5‐μL volume of bacterial resuspension was placed on a slide and air dried at room temperature. After Gram staining, bacterial cells were observed by light microscopy. Representative micrographs are shown for the strains of interest.

Although the optical density at 600 nm (OD_600_) of cells was very similar during growth in NYG medium, the cell morphology and CFU (colony‐forming units) numbers of the ΔnlpD mutant were significantly affected (Figs 2, S3), suggesting that *Xcc* NlpD plays a similar role in cell separation as *E. coli* NlpD. To obtain a better insight into the role of NlpD and the other putative LytM proteins in the cell division process, we examined the cell shape and size. For this, strains were cultured in the nutrient‐rich medium NYG overnight and bacterial cells were observed under a microscope after Gram staining. As illustrated in Fig. S4 (Supporting Information), most cells lacking NlpD (NK2522) or EnvC (NK0022) developed as unsegmented chains, whereas the other mutants tested displayed a similar phenotype to the wild‐type (single cells). This suggested that mutation of *nlpD* or *envC* resulted in a defect in cell separation and segmentation. Importantly, complementation partially reverted the ΔnlpD mutant towards the wild‐type phenotype (Fig. 2). Furthermore, the number of single cells in the CΔnlpD strain was greater than that of the mutant, although the CΔnlpD strain still formed some unsegmented chained cells (Fig. 2). In addition, the CFU number of the complementary strain CΔnlpD was significantly larger than that of the mutant ΔnlpD (Fig. S3). The *envC* mutant strain ΔenvC and the complemented strain CΔenvC exhibited a similar growth rate to the wild‐type in both NYG and MMX media. In addition, the cell shape and CFU number of ΔenvC could be restored to the levels of the wild‐type by *envC in trans* (Figs 2, S3). Although the reason why the phenotype of the mutant strain ΔnlpD could not be completely restored by *nlpD in trans* remains to be investigated, taken together, the data suggest that NlpD and EnvC are key factors for *Xcc* daughter cell separation, similar to the role of NlpD and EnvC in many other Gram‐negative bacteria.

### NlpD is required for *Xcc* T3S, but not T2S

It is well established that T2S and T3S are essential for *Xcc* virulence to host plants (Ryan *et al*., 2011). Therefore, to investigate whether the loss of virulence caused by the mutation of *nlpD* is associated with an impact on these secretion systems, we examined the ΔnlpD strain for impaired T2S and T3S. We assessed the activity of extracellular amylase, endoglucanase and protease as an indicator of T2S function. The results showed that ΔnlpD and the wild‐type produced similar activities of these enzymes (Table S3, see Supporting Information), indicating that mutation of *nlpD* did not affect T2S in *Xcc*.

Like many other bacterial pathogens, *Xcc* employs the T3SS to translocate effectors into host cells to overcome plant defences. Inactivation of *Xcc* T3S results in a loss of the ability to produce disease lesions in host plants and to elicit the HR in non‐host plants, such as the pepper cultivar ECW‐10R (Huang *et al*., 2009). To ascertain whether NlpD is required for *Xcc* T3S, we first determined the HR induction ability of the mutant ΔnlpD on the pepper cultivar ECW‐10R. The *hrcV*‐deficient mutant strain ΔhrcV was used as a negative control. HrcV is one of the core proteins composing the T3S apparatus and the mutant ΔhrcV is defective in T3S (Hartmann and Büttner, 2013). As shown in Fig. 1C, in contrast with the wild‐type strain 8004, which induced distinct HR symptoms at 8 h post‐inoculation, the mutant ΔnlpD could not elicit clear HR symptoms up to 16 h post‐inoculation. At 24 h post‐inoculation, a weak HR symptom was observed at the site of inoculation. These data indicate that deletion of *nlpD* leads to a delay in HR induction. The result was substantiated by an electrolyte leakage assay. ΔnlpD showed significantly lower electrolyte leakage relative to the wild‐type at 8 and 16 h post‐inoculation (Fig. 1D). As with virulence, the induction of HR and electrolyte leakage by the mutant ΔnlpD could be partly restored by *in trans* expression of the *nlpD* gene (i.e. CΔnlpD) (Fig. 1C,D). Interestingly, the *envC*‐deficient mutant induced HR symptoms and an electrolyte leakage similar to those caused by the wild‐type (Fig. 1C,D), suggesting that mutation of *envC* does not affect the function of the *Xcc* T3SS.

**Figure 3:**
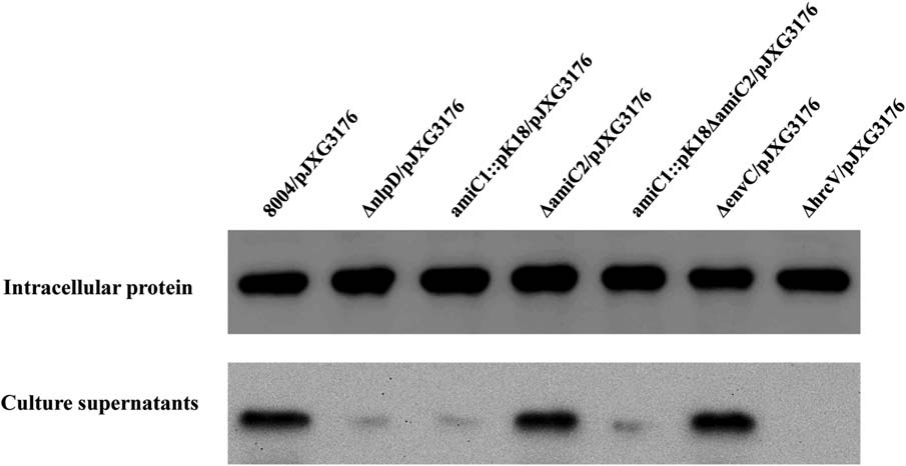
Mutation of *nlpD* or *amiC1* results in severely impaired type III secretion (T3S). The recombinant plasmid pJXG3176, which contains the T3S effector XC3176 encoding the open reading frame (ORF) without its stop codon, fused with 3 × Flag‐tag at its C‐terminus, was introduced into *Xanthomonas campestris* pv. *campestris* (*Xcc*) strains. The resulting recombinant strains were cultured in XCM2 medium for 12 h. The cells were harvested by centrifugation and the proteins in the cells and in the supernatant fraction were prepared. The proteins obtained were separated in a 12% sodium dodecylsulfate‐polyacrylamide gel electrophoresis (SDS‐PAGE) gel and analysed by Western blotting. 8004, ΔnlpD, amiC1::pK18, ΔamiC2, amiC1::pK18ΔamiC2, ΔenvC and ΔhrcV strains were tested.

To further validate the involvement of NlpD in the T3S of *Xcc*, we evaluated the secretion efficiency of the ΔnlpD strain. Our previous work identified a T3S effector (T3SE) from *Xcc* strain 8004, which was encoded by the ORF *XC_3176* and named XC3176 (Qian *et al*., 2005). To evaluate the efficiency of T3S, we measured the secretion of the T3SE XC3176 by Western blotting. To this end, the reporter plasmid, named pJXG3176, encoding an XC3176 T3SE fused with a 3 × Flag tag at the C‐terminus (i.e. XC3176‐Flag_3_) (see Experimental procedures), was introduced into the mutant strains ΔnlpD and ΔhrcV, as well as the wild‐type strain 8004, by triparental conjugation. The fusion protein XC3176‐Flag_3_ in strains ΔnlpD/pJXG3176, ΔhrcV/pJXG3176 and 8004/pJXG3176 (Table S2) was extracted and detected by Western blotting after cultivation in the T3S‐inducing minimal medium XCM2. As shown in Fig. 3, strain ΔnlpD/pJXG3176 produced a significantly lower amount of XC3176‐Flag_3_ protein in the culture supernatant compared with strain 8004/pJXG3176. As expected, XC3176‐Flag_3_ was undetectable in the culture supernatant from ΔhrcV/pJXG3176. However, all three strains produced a significant amount of intracellular XC3176‐Flag_3_ protein (Fig. 3). These results revealed that mutation of *nlpD* could lead to a severe defect in the *Xcc* T3S mechanism.

As mentioned above, *Xcc* T3S is encoded by a cluster of *hrp* genes consisting of *hrpA* to *hrpF* operons. It is possible that NlpD regulates the expression of *hrp* genes. To verify this possibility, we determined the expression level of the *hrp* operon (genes *hrpB* and *hrpF*) as well as two key *hrp* regulator genes, *hrpG* and *hrpX*, which positively regulate the expression of the *hrp* operon. For this purpose, the plasmid‐driven promoterless β‐glucuronidase (*gusA*) transcriptional fusion reporters of *hrpG*, *hrpX*, *hrpB* and *hrpF*, in which a DNA fragment containing the promoter region of *hrpG*, *hrpX*, *hrpB* or *hrpF* was fused to the promoterless *gusA* gene with its ribosome binding site (RBS) and cloned into the vector pLAFR6 (Table S2), were introduced by triparental conjugation to mutant ΔnlpD and the wild‐type strain 8004. The β‐glucuronidase (GUS) activities of the reporter strains obtained were determined after incubation in the minimal medium XCM2. The results showed that there was no significant difference in GUS activity between the mutant ΔnlpD and the wild‐type 8004 carrying the same reporter plasmid (Fig. S5, see Supporting Information), suggesting that NlpD is not involved in the regulation of *hrp* gene expression.

### NlpD is dispersed in cytoplasm, periplasm and outer membrane

It is known that the *E. coli* NlpD is a lipoprotein with an N‐terminal lipid moiety and must be localized to the outer membrane to properly control its activity (Tsang *et al*., 2017; Uehara *et al*., 2009). The lipid signal peptide serves as a membrane anchor, and so most Gram‐negative bacterial lipoproteins are retained in either the inner membrane or the outer membrane (Narita and Tokuda, 2016; Wilson and Bernstein, 2016). Domain structure analysis revealed that the *Xcc* NlpD also contains a lipid signal peptide with 22 amino acid residues at the N‐terminus (Table S1), suggesting that the *Xcc* NlpD is probably a lipoprotein as well. To determine the subcellular location of the NlpD protein in *Xcc*, a recombinant strain, named 8004_NlpD‐Flag_ (Table S2), was constructed (see below for details). The strain expresses a recombinant NlpD protein with a 3 × Flag tag at the C‐terminus (NlpD‐Flag). The total, periplasmic, cytoplasmic, inner membrane and outer membrane protein fractions of the recombinant strain 8004_NlpD‐Flag_ grown overnight in NYG medium were prepared. Western blotting analysis showed that the expressed recombinant NlpD‐Flag protein presented in all of the protein fractions except the inner membrane fraction (Fig. S6, see Supporting Information). These data suggest that, after synthesis in the cytoplasm, the NlpD protein is transported to the periplasm and then anchors to the outer membrane with the N‐terminus, supporting the prediction that *Xcc* NlpD is a lipoprotein.

### NlpD functions to bind to PG and modulate AmiC1 activity to degrade PG

**Figure 4:**
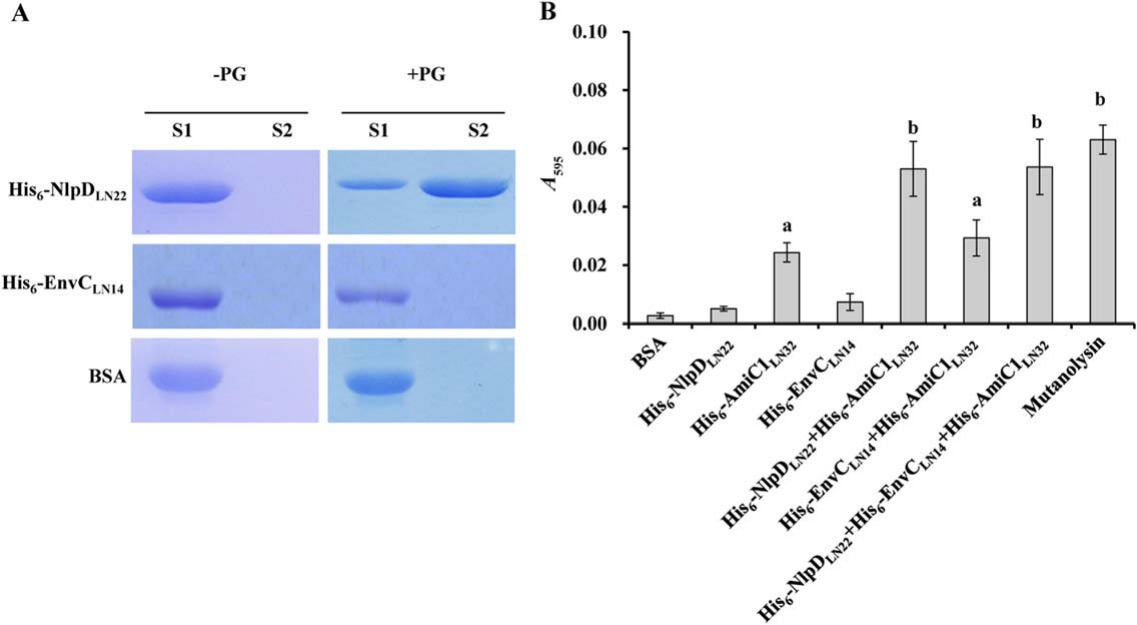
*Xanthomonas campestris* pv. *campestris* (*Xcc*) NlpD activates the peptidoglycan (PG) hydrolase activity of AmiC1. (A) His_6_‐NlpD_LN22_ can bind to PG *in vitro*. Purified His_6_‐NlpD_LN22_, His_6_‐EnvC_LN14_ and bovine serum albumin (BSA) proteins were mixed with PG in binding buffer and incubated for 2 h. The mixture was then centrifuged and the supernatant (S1) was collected and applied to sodium dodecylsulfate‐polyacrylamide gel electrophoresis (SDS‐PAGE) analysis; the pellet was resuspended in 2% SDS solution after washing with binding buffer and incubated for 1 h to allow the release of the protein from the predicted PG–protein complex. The resuspension was centrifuged and the supernatant (S2) was analysed by SDS‐PAGE. The gel was stained with Coomassie blue. –PG, without PG; +PG, with PG. (B) His_6_‐NlpD_LN22_ activates the PG hydrolase activity of His_6_‐AmiC1_LN32_ *in vitro*. Reactions containing remazol brilliant blue (RBB)‐labelled PG plus BSA (4 μm), His_6_‐NlpD_LN22_ (4 μm), His_6_‐AmiC1_LN32_ (4 μm), His_6_‐EnvC_LN14_ (4 μm) or mutanolysin (4 μm) were incubated at 37 °С for 16 h and then terminated by the addition of ethanol. Undigested PG was pelleted by centrifugation and the absorbance of the supernatants at 595 nm was determined. Data are the means ± standard deviations of triplicate measurements. The experiment was repeated three times and similar results were obtained. The different letters above each bar indicate significant differences at *P* = 0.05 by *t*‐test.

To understand the role played by NlpD in affecting T3S in *Xcc*, we began to biochemically characterize the protein. As described above, bioinformatics analysis revealed that *Xcc* NlpD carries a PG‐binding LysM domain, suggesting that the NlpD protein can bind to PG. To verify this suggestion, an *in vitro* pull‐down binding assay was performed. As illustrated in Fig. 4A, the S1 sample contained only a small amount of His_6_‐NlpD_LN22_ protein, whereas the S2 sample contained a large amount of His_6_‐NlpD_LN22_ protein (Fig. 4A), suggesting that most of the His_6_‐NlpD_LN22_ protein in the mixture was bound to PG to form a complex which was deposited in the pellet after the first centrifugation. These data confirm that the His_6_‐NlpD_LN22_ protein interacts physically with PG. Importantly, a control reaction using bovine serum albumin (BSA) protein showed that the BSA protein was present in the S1 sample, but not the S2 sample (Fig. 4A), indicating that the BSA protein did not bind PG and that interaction between PG and the His_6_‐NlpD_LN22_ protein is specific. His_6_‐EnvC_LN14_ protein was also tested. The result showed that it did not interact with PG in the test conditions (Fig. 4A).

To gain a further understanding of the function of *Xcc* NlpD, we assessed the ability of the purified His_6_‐NlpD_LN22_ protein to degrade *Xcc* PG using a dye release assay (Peters *et al*., 2013). Purified His_6_‐NlpD_LN22_ protein was incubated with *Xcc* PG labelled with the colorimetric dye remazol brilliant blue (RBB). NlpD degradation activity was determined by measuring the quantity of coloured dye present in the supernatant after stopping the reaction and centrifugation to deposit the intact PG. It was demonstrated that the mutanolysin of *Streptomyces globisporus* could degrade *Xanthomonas* PG (Erbs *et al*., 2008). In the assay, we used mutanolysin (Sigma, St. Louis, Missouri, USA) as a positive control and BSA protein as a negative control. No degradation activity was observed for either His_6_‐NlpD_LN22_ or BSA, even after 16 h of incubation, whereas mutanolysin displayed obvious PG degradation activity (Fig. 4B). This result indicates that *Xcc* NlpD cannot hydrolyse PG by itself *in vitro*.

Like *Xcc* NlpD, *E. coli* NlpD does not have PG hydrolytic activity by itself. The effect of *E. coli* NlpD on daughter cell separation is attained by specific activation of the amidase AmiC to hydrolyse PG (Uehara *et al*., 2009, 2010). The genome of *Xcc* strain 8004 encodes four *N*‐acetylmuramoyl‐l‐alanine amidases, two (XC1816 and XC2472) of which are amidase_3 family members and the others (XC2695 and XC3877) are amidase_2 family members (Qian *et al*., 2005). A SMART analysis revealed that both XC1816 and XC2472 possess an AMIN domain (Table S4, see Supporting Information), suggesting that both are homologues of *E. coli* AmiC. We therefore designated XC1816 and XC2472 as AmiC1 and AmiC2, respectively (Table S4). XC2695 and XC3877 were named AmpD and AmiD, respectively, based on both sequence homology and structural similarity (Table S4).

To investigate whether *Xcc* NlpD affects cell division in a mode similar to that of *E. coli* NlpD, we first constructed *Xcc* mutant strains defective in AmiC1 or AmiC2 and inspected the daughter cell separation of the mutants. As shown in Fig. 2, the *amiC1* mutant strain amiC1::pK18 formed unsegmented chained cells, indicating that *Xcc* AmiC1 is indispensable for regular daughter cell separation. The overwhelming majority of cells of the mutant strain lacking AmiC2 (ΔamiC2) were similar to those of the wild‐type, although some short chains consisting of three to four cells were observed (Fig. 2), suggesting that *Xcc* AmiC2 is not essential, but has a minor effect, on daughter cell separation. A strain deficient in both AmiC1 and AmiC2 (amiC1::pK18ΔamiC2) displayed a more severe chaining phenotype compared with the single mutants alone (Fig. 2), supporting the idea that AmiC2 plays some role in the separation of daughter cells, but is not critical. This was further supported by the observation that the amiC1::pK18ΔamiC2 mutant could be restored by complementation with *amiC1*, but not *amiC2*, expressed *in trans* (Fig. 2). This confirmed that AmiC1 plays a critical role in cell division. Next, we studied the function of AmiC1 on PG degradation. To achieve this, a recombinant 6 × His‐tagged truncated AmiC1 protein (His_6_‐AmiC1_LN32_) was created and overexpressed in *E. coli*. We also made an attempt to overproduce a recombinant 6 × His‐tagged truncated AmiC2 protein (His_6_‐AmiC2_LN23_) by the same means, but failed to obtain a soluble form of the fusion protein. The ability of AmiC1 to degrade PG *in vitro* was assessed using dye release assays. These experiments revealed that the His_6_‐AmiC1_LN22_ protein could only weakly degrade PG (Fig. 4B). However, the presence of His_6_‐NlpD_LN22_, but not His_6_‐EnvC_LN14_, could significantly enhance PG degradation by His_6_‐AmiC1_LN32_ (Fig. 4B). These results suggest that *Xcc* NlpD influences cell division by modulation of AmiC1 activity to hydrolyse PG, similar to *E. coli* NlpD. The mutant strain ΔenvC expressing *in trans* a recombinant gene encoding the His_6_‐EnvC_LN14_ protein (strain C_2_ΔenvC) showed the wild‐type phenotype (Fig. S7, see Supporting Information), suggesting that His_6_‐EnvC_LN14_ is functional, but cannot activate AmiC1.

### AmiC1 is essential for *Xcc* virulence and T3S

**Figure 5:**
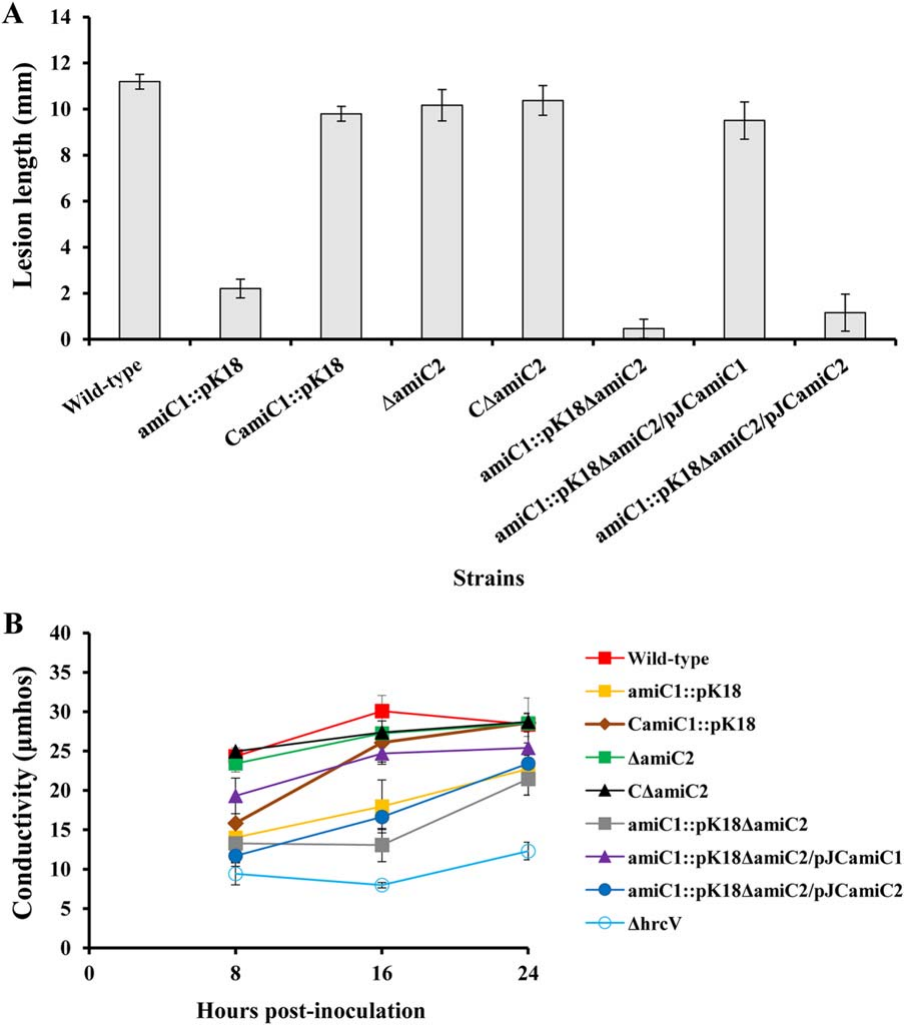
AmiC1 is essential for virulence and hypersensitive response (HR) induction by *Xanthomonas campestris* pv. *campestris* (*Xcc*). *Xcc* cells cultured overnight were washed and resuspended in double‐distilled H_2_O to an optical density at 600 nm (OD_600_) of 0.001 for the virulence test and 0.01 for the HR (electrolyte leakage) test. (A) Average lesion lengths caused by *Xcc* strains. Chinese radish (*Raphanus sativus*) leaves were inoculated by cutting leaves with scissors dipped in the bacterial suspensions. Lesion lengths were scored at 10 days post‐inoculation. Values represent means and standard deviations from 60 inoculated leaves in one experiment. The experiment was repeated three times with similar results. (B) Electrolyte leakage from pepper leaves inoculated with *Xcc* strains. Bacterial resuspension was infiltrated into the leaf mesophyll tissue of pepper (*Capsicum annuum* cv. ECW‐10R) with a blunt‐end plastic syringe. For each sample, three leaf discs were removed with a 0.7‐cm‐diameter cork borer from the infiltrated area. Three samples were taken for each measurement in each experiment. Results presented are from a representative experiment, and similar results were obtained in other experiments.

The above results demonstrate that *Xcc* NlpD affects cell division by activating AmiC1 PG degradation and is involved in virulence and T3S. It is possible that the influence of NlpD on virulence and T3S is also accomplished via the modulation of AmiC1 activity. If so, removal of AmiC1 should result in attenuated virulence and T3S. To assess this possibility, we first examined the virulence of the mutant strain amiC1::pK18. Indeed, the virulence of the mutant in the host plant Chinese radish was severely diminished. At 10 days post‐inoculation, the wild‐type strain caused disease symptoms with a mean lesion length of approximately 11 mm, whereas the mutant induced very weak disease symptoms with a mean lesion length of only about 2 mm (Fig. 5A). The HR induction ability of the mutant was then determined in the non‐host plant pepper cultivar ECW‐10R using an electrolyte leakage assay. As shown in Fig. 5B, the leaf tissues inoculated with the mutant had a much lower electrolyte leakage compared with those inoculated with the wild‐type, suggesting that AmiC1 is important for *Xcc* HR induction. Consistent with the effect on cell division, deletion of AmiC2 only slightly affected the virulence and electrolyte leakage (Fig. 5B). Furthermore, the deletion of AmiC2 in the AmiC1‐deficient background (amiC1::pK18ΔamiC2 double mutant) further depressed the virulence and electrolyte leakage (Fig. 5B). Interestingly, AmiC1, but not AmiC2, was able to restore the virulence of the amiC1::pK18ΔamiC2 double mutant (Fig. 5).

To further verify the idea that NlpD influences T3S via the activation of AmiC, the T3S efficiency of the mutant amiC1::pK18 was determined by Western blot analysis using the effector fusion protein XC3176‐Flag_3_. As with the previous experiments detailed above, the XC3176‐Flag_3_‐encoding plasmid pJXG3176 was introduced into the mutant strains amiC1::pK18, ΔamiC2 and amiC1::pK18ΔamiC2. The resulting recombinant strains amiC1::pK18/pJXG3176, ΔamiC2/pJXG3176 and amiC1::pK18ΔamiC2/pJXG3176 were used to examine the secretion of XC3176‐Flag_3_ protein by Western blot assay. As shown in Fig. 3, a large amount of XC3176‐Flag_3_ protein was present in the cells of all the strains tested and in the culture supernatants of the strain ΔamiC2/pJXG3176, but a very small amount was present in the supernatants of the strains amiC1::pK18/pJXG3176 and amiC1::pK18ΔamiC2/pJXG3176. These results suggest that AmiC1 is critical for T3S of *Xcc*. The extracellular amylase, endoglucanase and protease activities of the mutant strains amiC1::pK18, ΔamiC2 and amiC1::pK18ΔamiC2 were similar to those of the wild‐type (Table S3), indicating that AmiC1 and AmiC2 are not involved in *Xcc* T2S.

## Discussion

*N*‐Acetylmuramoyl‐l‐alanine amidases and their LytM activation factors are broadly distributed in bacteria, with members of this family in each species. In *E. coli*, the amidases AmiA, AmiB and AmiC affect cell division via the cleavage of PG bonding and their hydrolytic activities are stimulated by the LytM activation factors EnvC (AmiA and AmiB) and NlpD (AmiC) (Heidrich *et al*., 2001; Uehara *et al*., 2009, 2010). Here, we show, for the first time, that AmiC and its LytM family activator NlpD, as well as being important for cell separation, play a role in T3S and virulence in the plant pathogen *Xcc*. Unlike *E. coli*, *Xcc* does not possess AmiA and AmiB homologues, but encodes two proteins homologous to AmiC (AmiC1 and AmiC2) (Table S4). In addition to LytM factor NlpD, *Xcc* also encodes EnvC. Interestingly, *Xcc* strains lacking NlpD, EnvC or AmiC1 displayed a severe defect in daughter cell separation, whereas the AmiC2‐deficient mutant showed only a slight defect in cell division (Figs 2, S4). Purified recombinant proteins His_6_‐NlpD_LN22_ and His_6_‐EnvC_LN14_ could interact individually with purified *Xcc* PG *in vitro*, but could not degrade PG by themselves. In addition, the *in vitro* PG degradation activity of the purified His_6_‐AmiC1_LN32_ protein was weak, but could be strongly activated by His_6_‐NlpD_LN22_, but not His_6_‐EnvC_LN14_ (Fig. 4). Taken together, these observations suggest that the LytM factors EnvC and NlpD, as well as the amidase AmiC1, are essential for *Xcc* daughter cell separation and NlpD functions via the modulation of the amidase activity of AmiC1. These findings closely mirror the observations in *Vibrio cholerae*, which encodes only a single amidase, AmiB, and two LytM factors, EnvC and NlpD, both of which can activate AmiB to cleave PG and affect cell division (Möll *et al*., 2014).

Although *Xcc* encodes an EnvC homologue, its role in cell separation is still unclear. This work showed that the purified His_6_‐EnvC_LN14_ protein could neither degrade PG itself nor activate His_6_‐AmiC1_LN32_. Given the different phenotypes observed on daughter cell separation of the mutants deficient in EnvC or AmiC2, and the fact that His_6_‐EnvC_LN14_ could not activate His_6_‐AmiC1_LN32_, *Xcc* EnvC may influence cell division by an as yet unknown mechanism, which is probably independent of AmiC1, although we cannot exclude the possibility that *Xcc* EnvC can activate AmiC2. Mutation of *amiC2* resulted in a small portion of cells forming short chains and inactivation of *amiC2* in the *amiC1*‐deficient background potentiated the daughter cell separation defect. These results indicate that AmiC2 is also associated with cell division, but its influence is much weaker than that of AmiC1. Unfortunately, our attempt to study the potential enzymatic activity of AmiC2 failed because of our inability to express a soluble form of AmiC2 protein.

Although NlpD, AmiC1 and EnvC have a similar effect on cell division, their significance on virulence is very different. The strains lacking either NlpD or AmiC1 almost completely lost virulence, but the mutant lacking EnvC still demonstrated levels of virulence comparable with those of the wild‐type (Figs 1, 5, S1). These data, together with the observation that *nlpD*‐, *amiC1*‐ and *envC*‐deficient mutants displayed a chained cell phenotype, indicate that the formation of long‐chained cells does not impact the pathogenicity of *Xcc*. The fact that the mutants of *nlpD* and *amiC1*, but not *envC*, showed a severe defect in T3S suggests that the loss of virulence for *nlpD* and *amiC1* mutants is associated with deficiency in the T3SS rather than the defect in daughter cell separation. The bacterial T3S apparatus is a complex macromolecular nanomachine made up of more than 20 proteins, which can be divided into three parts: an extracellular pilus or needle‐like appendage, a membrane‐spanning basal body and the peripheral inner membrane cytoplasmic components. The basal body supports the pilus or needle appendage by anchoring it on the bacterial membranes (Abrusci *et al*., 2014; Büttner, 2012; Deng *et al*., 2017; Galán *et al*., 2014; Portaliou *et al*., 2016). Although great progress has been made in the understanding of the mechanism by which the bacterial cells assemble the T3S machinery, there are still some crucial issues to be addressed. One of the issues is how the basal body crosses the bacterial cell wall during the assembly process (Deng *et al*., 2017; Scheurwater and Burrows, 2011). It is understood that the PG mesh becomes an impediment to the basal body crossing and therefore needs to be remodelled during T3S assembly (Burkinshaw *et al*., 2015; Demchick and Koch, 1996; Meroueh *et al*., 2006). Given that, in the PG mesh, the first peptide of the peptide side‐chains is l‐alanine, which is attached to MurNAc in the glycan strands, the *N*‐acetylmuramoyl‐l‐alanine amidases are capable of releasing the peptide side‐chains from the MurNAc residue by specifically cleaving the amide bond between MurNAc and *L*‐alanine, which would free space (Frirdich and Gaynor, 2013; Vollmer *et al*., 2008). In *E. coli* and other bacteria, the enzymatic activities of the *N*‐acetylmuramoyl‐l‐alanine amidases are activated by LytM family regulators; our data confirm a similar system in *Xcc* by showing that AmiC1 is activated by the LytM factor NlpD. This fact, coupled with the observation that the *Xcc* mutant cells in the absence of either AmiC1 or NlpD were defective in T3S, suggests that the cleavage of the amide bond by AmiC1/NlpD is associated with T3S. Therefore, it is reasonable to deduce that the cleavage of the peptide side‐chain by AmiC1/NlpD is a crucial action to remodel the PG layer for facilitation of the T3S apparatus assembly in *Xcc*. This is also consistent with our observations showing that NlpD is dispersed in the cytoplasm, periplasm and outer membrane (Fig. S6).

This work also shows that the T2S system (T2SS) is regularly functional in the mutants lacking either AmiC1 or NlpD, as well as in the strains lacking both AmiC1 and AmiC2. This finding indicates that the effect of AmiC1/NlpD on the *Xcc* T3SS is specific. The T2SS is composed of more than a dozen proteins. The outer membrane secretin (GspD) is a core component of the T2S architecture, which penetrates the PG layer (Gu *et al*., 2017). It is thought that, in *Aeromonas* and *Vibrio* species, a complex formed by two different proteins (GspA and GspB) is involved in the modification of the PG layer to allow the assembly of the GspD secretin (for a review, see Gu *et al*., 2017). However, no direct evidence has been reported to date to show that a PG‐lytic enzyme is involved in T2SS assembly. There is no doubt that the cleavage of PG by the amidase enzymes must be controlled in a timely and spatially correct manner, otherwise it will be destructive to bacterial cells. Previous studies have demonstrated that, during cytokinesis in *E. coli*, PG hydrolysis at the division site leading to daughter cell separation is precisely regulated to avoid uncontrolled cell lysis. The amidases AmiB and AmiC, as well as their activators EnvC and NlpD, are recruited to the division site, and the localization of AmiC, AmiB and NlpD requires the essential divisome protein FtsN (Bernhardt and de Boer, 2003; Peters *et al*., 2013; Uehara *et al*., 2010). Of course, there are many unanswered questions, but one that is outstanding is: ‘What is the mechanism by which *Xcc* cells coordinate the cleavage of PG by AmiC1/NlpD during T3S apparatus assembly?’ This is a topic that merits further investigation.

## Experimental Procedures

### Bacterial strains, plasmids and growth conditions

The bacterial strains and plasmids used are described in Table S2. *Escherichia coli* strains were grown in Luria–Bertani (LB) medium (Miller, 1972) at 37 °C, and *Xcc* strains were grown in NYG rich medium (3 g/L yeast extract, 5 g/L peptone, 20 g/L glycerol, pH 7.0) (Daniels *et al*., 1984a), XCM2 induction medium (20 mm succinic acid, 0.15 g/L casamino acids, 7.57 mm (NH_4_)_2_SO_4_, 0.01 mm MgSO_4_, 60.34 mm K_2_HPO_4_, 33.07 mm KH_2_PO_4_, pH 6.6) and MMX minimal medium (5 g/L glucose, 1 g/L sodium citrate, 2 g/L (NH_4_)_2_SO_4_, 0.2 g/L MgSO_4_, 4 g/L K_2_HPO_4_, 6 g/L KH_2_PO_4_, pH 7.0) (Daniels *et al*., 1984b) at 28 °C. Antibiotics were used at the following final concentrations: ampicillin (Amp), 100 μg/mL; gentamycin (Gm), 10 μg/mL; kanamycin (Kan), 25 μg/mL; rifampicin (Rif), 50 μg/mL; spectinomycin (Spc), 50 μg/mL; tetracycline (Tc), 15 μg/mL for *E. coli* and 5 μg/mL for *Xcc*.

### Determination of the transcriptional start site

The transcriptional start site of the gene *XC_2522* encoding the LytM protein NlpD was determined by 5′ RACE analysis (Fig. S2). Total cellular RNA was extracted by RNAiso Blood (TaKaRa, Dalian, China) and treated with RQ1 RNase‐free DNase (Promega, Madison, Wisconsin, USA), followed by a second purification. The cDNA fragments were obtained using the 5′ RACE kit (Invitrogen, Carlsbad, California, USA). RNA was reverse transcribed using the *XC_2522* sequence‐specific primer 2522GSP1 (Table S5, see Supporting Information). An anchor sequence was then added to the 3′ end of the cDNA using terminal deoxynucleotide transferase, followed by direct amplification of tailed cDNA using the nested gene‐specific primers 2522GSP2 and 2522GSP3 (Table S5) and the anchor‐specific primer provided. Polymerase chain reaction (PCR) products were then cloned into the pGEM‐T Easy vector (Promega) and sequenced (Table S6, see Supporting Information).

### Construction of strains for protein expression

For the overproduction of a truncated NlpD protein lacking the N‐terminal 22‐amino‐acid signal peptide, a 720‐bp fragment was amplified using the primer pairs E2522‐F(B)/E2522‐R(E) (Table S5) designed according to the sequence of the *nlpD* ORF *XC_2522* reported in the *Xcc* strain 8004 (Qian *et al*., 2005). The amplified fragments were cloned into the vector pET30a to generate the recombined plasmid pET‐NlpD_LN22_. The plasmid pET‐NlpD_LN22_ was introduced into the *E. coli* strain BL21(DE3) to create the strain His_6_‐NlpD_LN22_ (Table S2), which was used for protein production. The strains His_6_‐EnvC_LN14_, His_6_‐AmiC1_LN32_ and His_6_‐AmiC2_LN23_ (Table S2) for the overproduction of 6 × His‐tagged truncated EnvC, AmiC1 and AmiC2 proteins, respectively, were constructed, and expressed in *E. coli* in accordance with the above method.

### Mutant construction and complementation

The mutants of the genes (*XC_0022*, *XC_0463*, *XC_0921*, *XC_1250*, *XC_1354*, *XC_1857*, *XC_2522*, *XC_3502*, *XC_3926* and *XC_4282*) encoding proteins with a LytM domain, named NK0022, NK0463, NK0921, 182E08, NK1354, 032B12, NK2522, 209A07, NK3926 and 024A02, respectively (Table S2), were obtained from the mutant library of the *Xcc* strain 8004, which was constructed by transposon (Tn5*gusA5*) insertion or homologous suicide plasmid (pK18mob) integration mutagenesis in our laboratory.

To construct the deletion mutant of *nlpD*, the region from 718 bp upstream to 722 bp downstream of the *XC_2522* ORF was amplified by the primer pairs D2522‐LF(B)/D2522‐LR(X) and D2522‐RF(X)/D2522‐RR(H) (Table S5), and fused into the suicide plasmid pk18mobsacB to create the recombinant plasmid pKD2522. The plasmid pKD2522 was transferred into the *Xcc* wild‐type strain 8004 by triparental conjugation, followed by the selection of colonies on NYG plates containing Rif and Kan. The *nlpD* deletion mutant was obtained by further selection on an NYG plate supplemented with Rif and 10% sucrose. The mutant colonies were then checked for Kan sensitivity and were further confirmed by PCR. The confirmed mutant was named ΔnlpD. Other deletion mutants were constructed in accordance with this method.

To construct the plasmid integration mutant of *amiC1* (ORF *XC_1816*), a 378‐bp internal fragment of *XC_1816* was amplified by the primer set NK1816‐F(B)/NK1816‐R(X) (Table S5). The confirmed sequenced DNA fragment was cloned into the suicide plasmid pK18mob (Table S2) to create the recombinant plasmid pK1816 (Table S2). The plasmid pK1816 was introduced from *E. coli* DH5*α* into the *Xcc* wild‐type strain 8004 by triparental conjugation. The transconjugants were screened on a NYG plate supplemented with Rif and Kan, and the *amiC1* mutant obtained was confirmed by PCR and named amiC1::pK18 (Table S2). Confirmation PCR was performed using the total DNA of the *amiC1* mutant cells obtained as the template and the primers pKmob18Con (located in pK18mob) and D1816RR (located downstream of the *amiC1* gene) (Table S5). The *amiC2* deletion mutant ΔamiC2 (Table S2) was constructed using the above deletion mutant construction method. The *amiC1* and *amiC2* double mutant amiC1::pK18ΔamiC2 (Table S2) was constructed in the background of the mutant ΔamiC2.

For complementation of the *nlpD* mutant, a 1359‐bp DNA fragment containing the *nlpD* coding region and extending from 491 bp upstream of the 5′ end to 40 bp downstream of the 3′ end of the ORF was amplified using the primer set C2522‐F(B)/C2522‐R(H) (Table S5), and the amplified DNA fragment was cloned into the plasmid pLAFR6 (Table S2) to generate the recombinant plasmid pLCnlpD (Table S2). The recombinant plasmid was transferred into the deletion mutant of *nlpD* by triparental conjugation, resulting in the complemented strain CΔnlpD (Table S2). Other complementary strains were constructed in accordance with the above method, except for the use of pLAFRJ instead of pLAFR6.

### Virulence assay

The virulence of *Xcc* strains in Chinese radish manshenhong was tested by the leaf clipping method, as described previously (Dow *et al*., 2003). The bacterial cultures were grown overnight at 28 °С with shaking at 200 rpm in NYG medium and the concentration was adjusted to an OD_600_ of 0.001. The Chinese radishes were planted in a glasshouse with natural light and temperatures of 25 to 30 °С. Five‐week‐old seedlings with four fully expanded leaves were inoculated by clipping the vein endings of the expanded leaves with sterilized scissors dipped in the bacterial cultures. At least 60 leaves were inoculated for each strain, and the experiment was repeated three times independently. After inoculation, the plants were maintained in 80% humidity for the first 24 h, and then in the conditions described above. The lesion lengths of the inoculated leaves were measured at 10 days post‐inoculation, and the data were analysed by *t*‐test.

### Detection of HR

For HR test, seedlings of the *Xcc* non‐host plant pepper (*Capsicum annuum* cv. ECW‐10R) were grown in a glasshouse with a 12‐h day and night cycle with illumination by fluorescent lamps at temperatures of 25–28 °C. The bacterial cells of *Xcc* strains from overnight cultures were washed and resuspended in sterile water. The bacterial resuspensions were diluted to an OD_600_ of 0.01 in double‐distilled H_2_O and infiltrated into the pepper leaf tissues at the stage of four fully expanded leaves using a needleless syringe. After infiltration, the plants were grown at 28 °C and 80% relative humidity. HR symptoms were photographed at 8, 16 and 24 h post‐inoculation. At least three plants were inoculated in each experiment, and each experiment was repeated at least three times. For the electrolyte leakage assays, measurements were carried out as described previously (Castañeda *et al*., 2005). Essentially, for each sample, three leaf discs were removed with a 0.7‐cm‐diameter cork borer, submerged in 10 mL of double‐distilled H_2_O and vacuum infiltrated. Then, the net leakage after 1 h was measured with a conductivity meter. Three samples were taken for each measurement in each experiment, and the experiments were repeated at least three times.

### Microscopic assays

For the light microscopy assay, bacterial cells of *Xcc* strains from overnight cultures were diluted in distilled water. The diluted bacterial suspension was spread on slides, stained with Gram staining solution and observed using an Olympus microscope BX41 (Tokyo, Japan).

### Flag epitope‐tagging of NlpD

For Flag‐tagging of NlpD, two fragments containing 798 bp upstream and 730 bp downstream, respectively, of the *nlpD* termination codon were amplified and *Eco*RI, *Bam*HI, *Xba*I and *Hin*dIII restriction sites were introduced. The amplified fragments and 3 × Flag oligonucleotides (custom made by Invitrogen) with *Bam*HI and *Xba*I restriction sites were fused together and cloned into the *Eco*RI and *Hin*dIII restriction sites of the suicide plasmid pK18mobsacB. The recombinant plasmid obtained was introduced into the *Xcc* wild‐type 8004 by triparental conjugation and the sequence of the wild‐type *nlpD* gene on the chromosome was replaced with *nlpD*‐3 × Flag. The resulting strain was named 8004_NlpD‐Flag_ and was used to investigate the subcellular localization of NlpD (NlpD‐Flag) by Western blot analysis. For this purpose, protein samples were separated in 12% sodium dodecylsulfate‐polyacrylamide gel electrophoresis (SDS‐PAGE) gel and transferred onto PVDF (polyvinylidene difluoride) membrane (Millipore, Billerica, Massachusetts, USA). After blocking, the 1 : 2000 diluted anti‐Flag‐tag rabbit monoclonal antibody (Sigma) was used as the primary antibody, and the 1 : 5000 diluted horseradish peroxidase‐conjugated goat anti‐rabbit immunoglobulin G (IgG) (Pierce, Rockford, Illinois, USA) was used as secondary antibody.

### Preparation of total, periplasmic and membrane proteins

The bacterial total and periplasmic proteins were prepared by the method reported previously (Zang *et al*., 2007). Preparation of the outer and inner membrane proteins was carried out as described by Chen *et al*. (2010). Briefly, 100 mL of *Xcc* cells were grown in NYG medium at 28 °С overnight and collected by centrifugation at 3 488 *g* at 4 °C for 10 min. The cells were suspended in cold TM buffer (10 mm Tris, pH 8.0, containing 8 mm MgSO_4_), washed three times, resuspended in 4.0 mL of the same buffer and broken by sonication. The unbroken cells and the cell debris were removed by centrifugation at 14 000 *g* at 4 °C for 30 min. The supernatants were further centrifuged at 135 000 *g* at 4 °C for 1 h and the supernatants containing cytoplasmic and periplasmic proteins were retained. The pellets containing membranes and ribosomes were suspended in 1.0 mL of cold TM buffer by gentle aspiration and ejection using a 25‐gauge needle attached to a 1‐mL syringe, and the resuspended samples were centrifuged at 135 000 *g* as before. The pellets were rinsed with 1.0 mL of cold TM buffer, resuspended in 3.9 mL of 0.25% (w/v) sarkosyl and loaded into 3.9‐mL ultracentrifuge tubes. After incubation at room temperature for 1 h, the tubes were centrifuged at 135 000 *g* for 1 h, as above. The supernatants containing the sarkosyl‐soluble inner membrane fraction were retained. The sarkosyl‐insoluble pellets containing the outer membrane fraction were washed twice with 1.0 mL of 0.25% (w/v) sarkosyl, incubated at room temperature for 1 h and centrifuged at 135 000 *g*, as described above. The pellets containing the outer membrane fraction were suspended in 40 μL of cold TM buffer.

### Purification of recombinant proteins

The overnight cultured *E. coli* strains for protein overexpression were transferred into 200 mL of LB medium and cultivated at 37 °C to an OD_600_ of 0.4. Then, 0.5 mm IPTG (Isopropyl β‐D‐thiogalactoside) was added and the strains were cultivated at 18 °C for 5 h. In particular, the strain His_6_‐EnvC_LN14_ was induced with 0.125 mm IPTG and grown at 18 °C for 3 h. The cells were harvested and resuspended in 10 mL of lysis buffer (20 mm Tris, 500 mm NaCl, 10 mm imidazole, pH 8.0). After ultrasonication, the cells were broken and the cell debris was removed by centrifugation at 12 000 *g* for 15 min at 4 °C. The supernatant was incubated with Ni‐agarose resin (CWBIO, Beijing, China) pre‐equilibrated with lysis buffer for 1 h at 4 °C. Then, the column was washed with 50 mL of wash buffer (20 mm Tris, 500 mm NaCl, 20 mm imidazole, pH 8.0). The recombinant proteins were eluted with elution buffer (20 mm Tris, 500 mm NaCl, 250 mm imidazole, pH 8.0). The imidazole was removed by ultrafiltration in PBS (10 mm Na_2_HPO_4_, 2 mm KH_2_PO_4_, 137 mm NaCl and 2.7 mm KCl, pH 7.4) with 10% glycerol. The proteins were detected in 12% SDS‐PAGE gel and stained with Coomassie blue.

### Purification of PG

The PG of *Xcc* was extracted and purified from dried cells as described previously (Glauner, 1988; Kuhner *et al*., 2014). Briefly, 300 mL of *Xcc* overnight culture was suspended in ice‐cold water and added drop‐wise to boiling 8% SDS and boiled for 30 min. After cooling to room temperature, the SDS‐insoluble material was collected by centrifugation. The pellet was washed several times with double‐distilled H_2_O until no SDS could be detected. Five millilitres of solution B (15 μg/mL DNase and 60 μg/mL RNase in 0.1 m Tris/HCl, pH 6.8) were added and incubated for 60 min at 37 °C in a shaker. Then, 5 mL of solution C (50 μg/mL trypsin in double‐distilled H_2_O) were added and incubated for an additional 60 min at 37 °C. The suspension was incubated at 100 °C for 3 min to inactivate the enzymes before centrifugation (5 min at 9 600 *g*). After washing with double‐distilled H_2_O, 1 m HCl was added and incubated for 4 h at 37 °C in a shaker. The PG in the suspension was spun down (5 min at 9 600 *g*) and resuspended in 1 mL of double‐distilled H_2_O after washing several times with double‐distilled H_2_O until the pH was 5–6. The purified PG can be stored at −20 °C.

### PG binding assay

The PG binding experiment was carried out as described by Rocaboy *et al*. (2013). Ten micrograms of purified protein were incubated for 2 h at room temperature with 10 μL of purified PG in binding buffer (30 mm Tris, pH 6.8, 50 mm NaCl, 10 mm MgCl_2_) in a total volume of 100 μL. The sample was centrifuged for 20 min at 14 000 *g* and the supernatant (S1) was analysed by 12% SDS‐PAGE. The pellet was washed twice with 150 μL of binding buffer and then resuspended in 40 μL of 2% SDS solution and incubated for 1 h at room temperature. The resuspension was centrifuged for 20 min at 14 000 *g* and the supernatant (S2) was analysed by SDS‐PAGE. The protein in SDS‐PAGE was determined by staining with Coomassie blue.

### Preparation of PG sacculi labelled with RBB

PG sacculi were labelled with RBB as described previously (Peters *et al*., 2013). The sacculi were incubated with 20 mm RBB (Sigma) in 0.25 m NaOH overnight at 37 °C. Then, the preparation was neutralized with HCl, and RBB‐labelled sacculi were pelleted by centrifugation for 20 min at 12 000 *g*. The labelled sacculi were washed several times with water until the supernatant was clear. The final pellet was resuspended in water and stored at 4 °C.

### PG cleavage assay

The PG hydrolysis activity of proteins was detected as described previously (Peters *et al*., 2013). RBB‐labelled sacculi (10 μL) were incubated with protein (4 μm) at 37 °C in 60 μL of PBS buffer for 16 h and 30 μL of ethanol were added to terminate the reaction, followed by centrifugation at 21 000 *g* for 20 min at room temperature. An Epoch 2 Microplate Spectrophotometer (BioTek, Winooski, Vermont, USA) was used to measure the absorbance of the supernatant at 595 nm.

### GUS activity assay

The overnight cultured *Xcc* strains in NYG medium were collected and cultivated for 20 h after suspension in XCM2 to an OD_600_ of 0.1. For determination of the total GUS activity, 1 mL of culture for each strain was mixed with 60 μL of methylphenol and vortexed. The supernatant was then taken for GUS activity assay. The GUS activity assay was performed by measurement of the OD_415_ using ρ‐nitrophenyl‐β‐d‐glucuronide as substrate, as described previously (Jefferson *et al*., 1986).

### Detection of *Xcc* T3S efficiency by Western blot analysis

To assess the T3S efficiency of *Xcc* strains, a recombinant plasmid, named pJXG3176 (Table S2), was constructed by cloning the T3SE XC3176‐encoding ORF without its stop codon into the vector pJXG, which was generated by cloning a DNA fragment containing the 3 × Flag‐tag coding sequence into the *Pst*I and *Hin*dIII restriction sites of pLAFRJ (Table S2). In pJXG3176, the 3′ end of the XC3176‐encoding ORF was fused to the 3 × Flag‐tag coding sequence to ensure the expression of an XC3176 protein with a 3 × Flag‐tag at its C‐terminus. The recombinant plasmid pJXG3176 was introduced into *Xcc* strains by triparental conjugation. The recombinant strains obtained were cultured in NYG medium overnight and cells were collected by centrifugation. After adjusting to OD_600_ = 0.1, the cells were transferred into *hrp*‐inducing medium XCM2 and incubated for 12 h. The cells were harvested by centrifugation at 3 488 *g* for 15 min. The proteins in the cells were isolated by resuspending the pellet in PBS buffer to OD_600_ = 0.2 and ultrasonicating for 1 min, followed by filtration. The proteins secreted in the supernatant fraction were filtered, precipitated with chilled trichloroacetic acid to a final concentration of 10% on ice overnight, washed with acetone and resuspended in rehydration solution (7 m urea, 2 m thiourea, 2% CHPAS (3‐((3‐Cholamidopropyl)dimethylammonium)‐1‐propanesulfonate). The proteins obtained were separated in 12% SDS‐PAGE gel and analysed by Western blotting as described above.

### Extracellular enzyme activity assay

The activities of the extracellular enzymes amylase, endoglucanase and protease were assessed quantitatively as described previously (Chao *et al*., 2008). Briefly, for endoglucanase activity quantification, 10 μL of cytoplasmatic extract was added to 200 μL of the indicated buffer containing 1% (w/v) carboxymethylcellulose (CMC). Reactions were carried out for 30 min at 28 °C. The released reducing sugars were measured as d‐glucose equivalents. One unit (U) of endoglucanase activity was defined as the amount of enzyme releasing 1 μmol of reducing sugar per minute. Amylase activity quantification was conducted in the same manner as the endoglucanase measurement, except that the substrate 1% (w/v) CMC was replaced by 1% (w/v) starch solution. The quantification of protease activity was performed as described by Swift *et al*. (1999).

## Supporting information

Additional Supporting Information may be found in the online version of this article at the publisher's website:

**Fig. S1** Results of leaf clipping assay to determine virulence in selected *Xanthomonas campestris* pv. *campestris* (*Xcc*) strains. The virulence of the mutant strains lacking a putative LytM protein was determined by leaf clipping assay in the host plant Chinese radish. Lesion lengths were scored at 10 days post‐inoculation. Values are the means ± standard deviations from three repeats, each with 60 leaves.

**Fig. S2** Genetic and physical map of the *nlpD* (*XC_2522*) gene in the genome of the *Xanthomonas campestris* pv. *campestris* (*Xcc*) strain 8004. The genetic positions and orientations of the genes *XC_2521*, *XC_2523*, *XC_2524* and *XC_2525* are shown: arrows indicate the length, location and orientation of the genes; lines between arrows indicate intergenic sequences. The transcriptional start site of *nlpD* determined in this study is indicated by a square. The putative −10 region, −35 region, Shine–Dalgarno (SD) sequence and the translational start codon (ATG) are displayed.

**Fig. S3** Growth characteristics of *Xanthomonas campestris* pv. *campestris* (*Xcc*) strains. An overnight culture [optical density at 600 nm (OD_600_) ≈ 1.0] of *Xcc* strains was inoculated into 10 mL of the nutrient‐rich medium NYG (A) or the minimal medium MMX (B), adjusted to an OD_600_ of 0.01 (for NYG) or 0.05 (for MMX) and then incubated at 28 °C with 200 rpm. The bacterial density was determined by measuring the OD_600_ values at several time points post‐inoculation (a, c). The cell number in the culture was determined using the dilution plate counting method, and the number of colony‐forming units (CFUs) was counted after incubation at 28 °C for 2 days (b, d). Data displayed are the means ± standard deviations from three replicates. The experiment was repeated three times and similar results were obtained.

**Fig. S4** *Xanthomonas campestris* pv. *campestris* (*Xcc*) *XC_2522* (*nlpD*) and *XC_0022* (*envC*) have a crucial effect on cell separation. Bacterial cells of *Xcc* strains cultured overnight in NYG medium were collected by centrifugation at 13 800 *g* for 30 s and resuspended in double‐distilled H_2_O. Five microlitres of bacterial resuspension were placed on a slide and dried at room temperature. After Gram's staining, bacterial cells were observed by light microscopy. Representative micrographs are shown.

**Fig. S5** Mutation of *nlpD* does not affect the expression of *hrp* genes. The β‐glucuronidase (GUS) activities of *hrpG*, *hrpX*, *hrpB* and *hrpF* promoter‐*gusA* reporters in *nlpD* deletion (ΔnlpD) and wild‐type (*Xcc* 8004) backgrounds were determined. *Xanthomonas campestris* pv. *campestris* (*Xcc*) strains were cultured in the minimal medium XCM2 for 20 h, and the GUS activities were then determined by measurement of the absorbance at 415 nm using ρ‐nitrophenyl‐β‐d‐glucuronide as substrate. Data are the means ± standard deviations of triplicate measurements. The experiment was repeated three times, and similar results were obtained.

**Fig. S6** Subcellular localization of *Xanthomonas campestris* pv. *campestris* (*Xcc*) NlpD by Western blot analysis. The bacterial total and periplasmic proteins, as well as the outer and inner membrane proteins, were prepared from 100 mL of *Xcc* cells grown in NYG medium overnight. Thirty micrograms of each protein sample were separated by sodium dodecylsulfate‐polyacrylamide gel electrophoresis (SDS‐PAGE) and transferred to a polyvinylidene difluoride (PVDF) membrane. Anti‐Flag‐tag was used to detect the presence of the recombinant protein NlpD‐Flag. NlpD‐Flag, protein from recombinant strain 8004_NlpD‐Flag_; WT, protein from the wild‐type strain 8004.

**Fig. S7** The mutant strain ΔenvC expressing *in trans* a recombinant gene encoding His_6_‐EnvC_LN14_ protein (strain C2ΔenvC) shows the wild‐type phenotype. Bacterial cells of *Xcc* strains cultured overnight in NYG medium were collected by centrifugation at 13 800 *g* for 30 s and resuspended in double‐distilled H_2_O. Five microlitres of bacterial resuspension were placed on a slide and dried at room temperature. After Gram staining, bacterial cells were observed by light microscopy. Representative micrographs are shown.

**Table S1** The predicted LytM proteins in *Xanthomonas campestris* pv. *campestris* (*Xcc*) and their homologues in *Escherichia coli*.

**Table S2** Bacterial strains and plasmids used in this work.

**Table S3** Quantification of the extracellular enzymes produced by *Xanthomonas campestris* pv. *campestris* (*Xcc*) strains.

**Table S4** The predicted *N*‐acetylmuramoyl‐l‐alanine amidases in *Xanthomonas campestris* pv. *campestris* (*Xcc*) and their homologues in *Escherichia coli*.

**Table S5** Primers used in this work.

**Table S6** 5′ Rapid amplification of cDNA ends (5′ RACE) sequencing result.
