# Supplementary figures and images for: Peptidoglycan hydrolysis mediated by the amidase AmiC and its LytM activator NlpD is critical for cell separation and virulence in the phytopathogen Xanthomonas campestris
Source: Mol Plant Pathol. 2018 Feb 1;19(7):1705–18. doi: 10.1111/mpp.12653 (PMC6638016; doi:10.1111/mpp.12653)

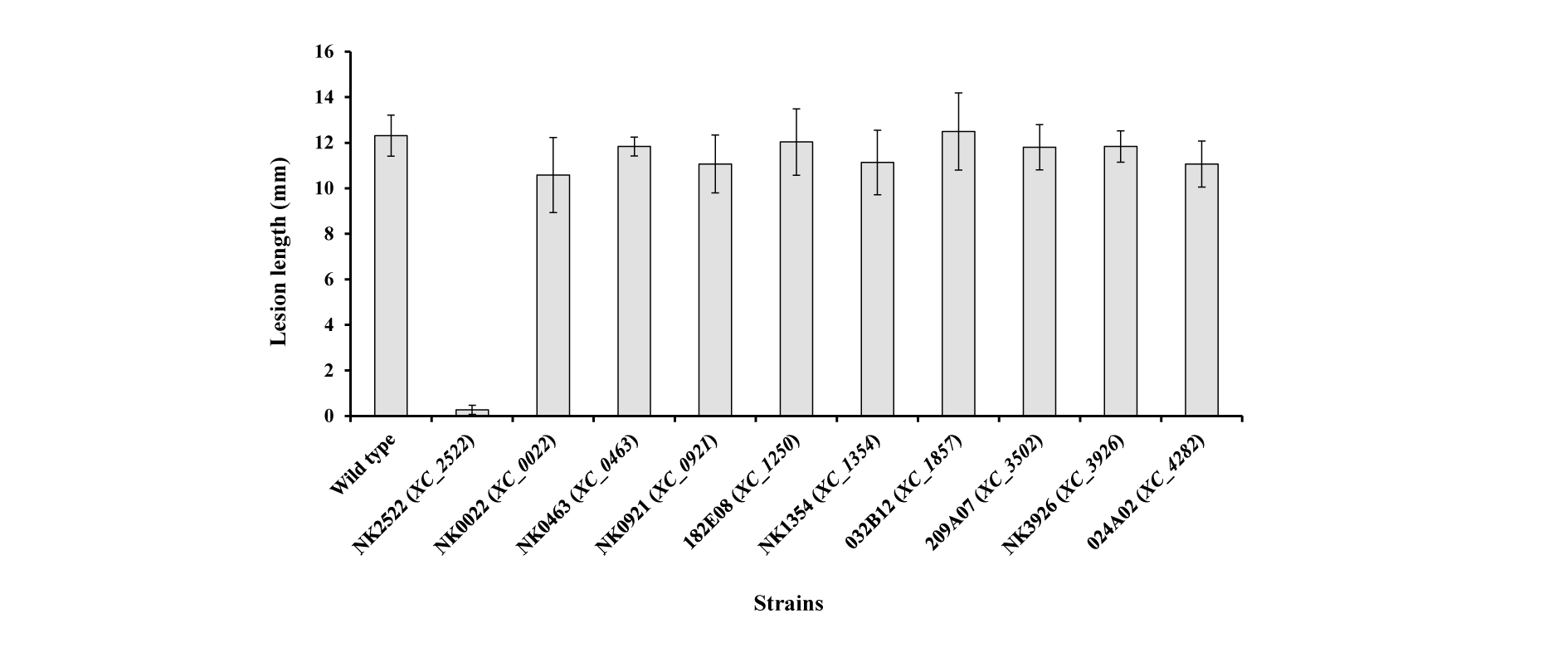

Supplement: Supplementary file 1 — Fig. S1 Results of leaf clipping assay to determine virulence in selected Xanthomonas campestris pv. campestris (Xcc) strains. The virulence of the mutant strains lacking a putative LytM protein was determined by leaf clipping assay in the host plant Chinese radish. Lesion lengths were scored at 10 days post‐inoculation. Values are the means ± standard deviations from three repeats, each with 60 leaves. [file MPP-19-1705-s001.tif]

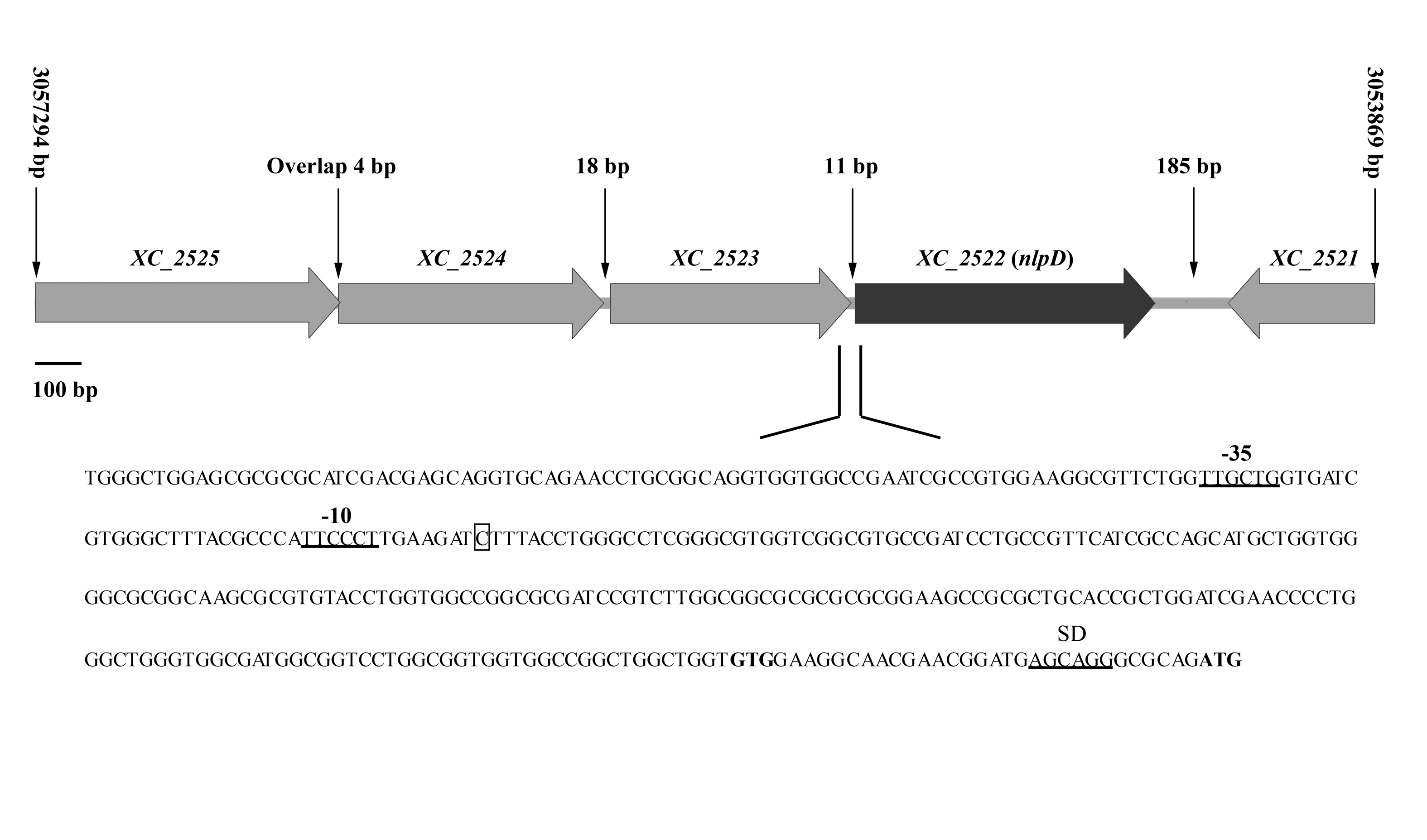

Supplement: Supplementary file 2 — Fig. S2 Genetic and physical map of the nlpD (XC_2522) gene in the genome of the Xanthomonas campestris pv. campestris (Xcc) strain 8004. The genetic positions and orientations of the genes XC_2521, XC_2523, XC_2524 and XC_2525 are shown: arrows indicate the length, location and orientation of the genes; lines between arrows indicate intergenic sequences. The transcriptional start site of nlpD determined in this study is indicated by a square. The putative −10 region, −35 region, Shine–Dalgarno (SD) sequence and the translational start codon (ATG) are displayed. [file MPP-19-1705-s002.tif]

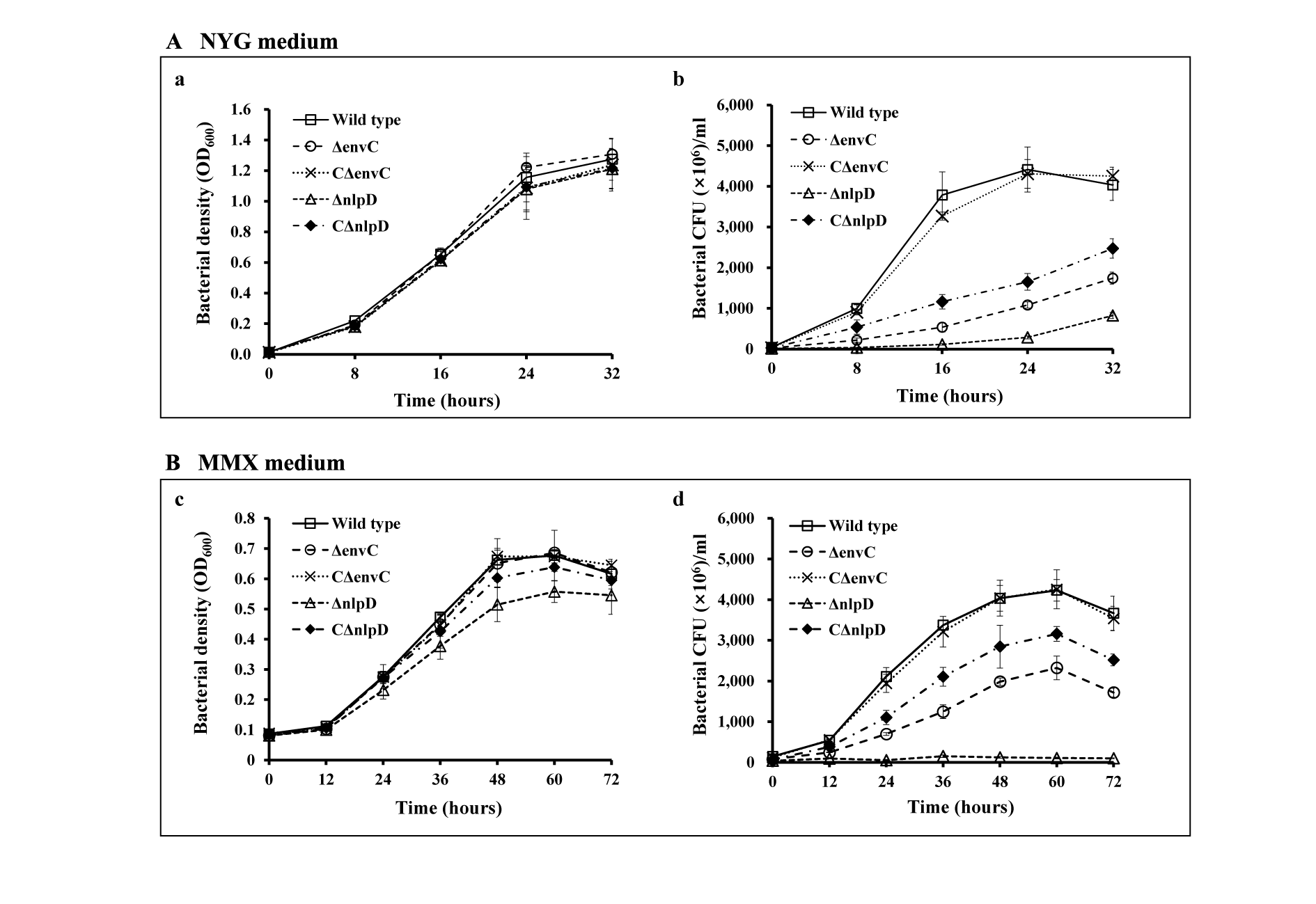

Supplement: Supplementary file 3 — Fig. S3 Growth characteristics of Xanthomonas campestris pv. campestris (Xcc) strains. An overnight culture [optical density at 600 nm (OD600) ≈ 1.0] of Xcc strains was inoculated into 10 mL of the nutrient‐rich medium NYG (A) or the minimal medium MMX (B), adjusted to an OD600 of 0.01 (for NYG) or 0.05 (for MMX) and then incubated at 28 °C with 200 rpm. The bacterial density was determined by measuring the OD600 values at several time points post‐inoculation (a, c). The cell number in the culture was determined using the dilution plate counting method, and the number of colony‐forming units (CFUs) was counted after incubation at 28 °C for 2 days (b, d). Data displayed are the means ± standard deviations from three replicates. The experiment was repeated three times and similar results were obtained. [file MPP-19-1705-s003.tif]

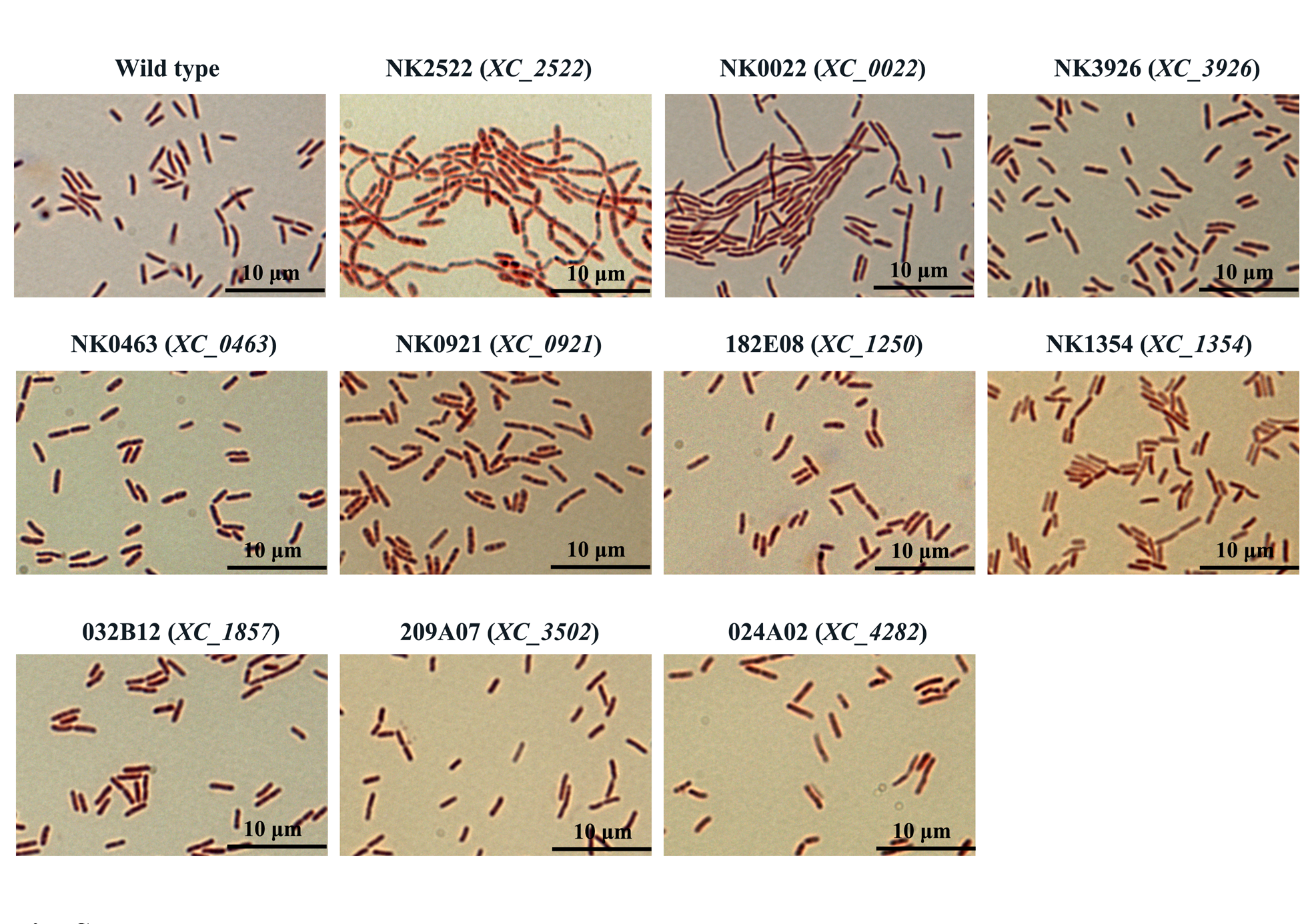

Supplement: Supplementary file 4 — Fig. S4 Xanthomonas campestris pv. campestris (Xcc) XC_2522 (nlpD) and XC_0022 (envC) have a crucial effect on cell separation. Bacterial cells of Xcc strains cultured overnight in NYG medium were collected by centrifugation at 13 800 g for 30 s and resuspended in double‐distilled H2O. Five microlitres of bacterial resuspension were placed on a slide and dried at room temperature. After Gram's staining, bacterial cells were observed by light microscopy. Representative micrographs are shown. [file MPP-19-1705-s004.tif]

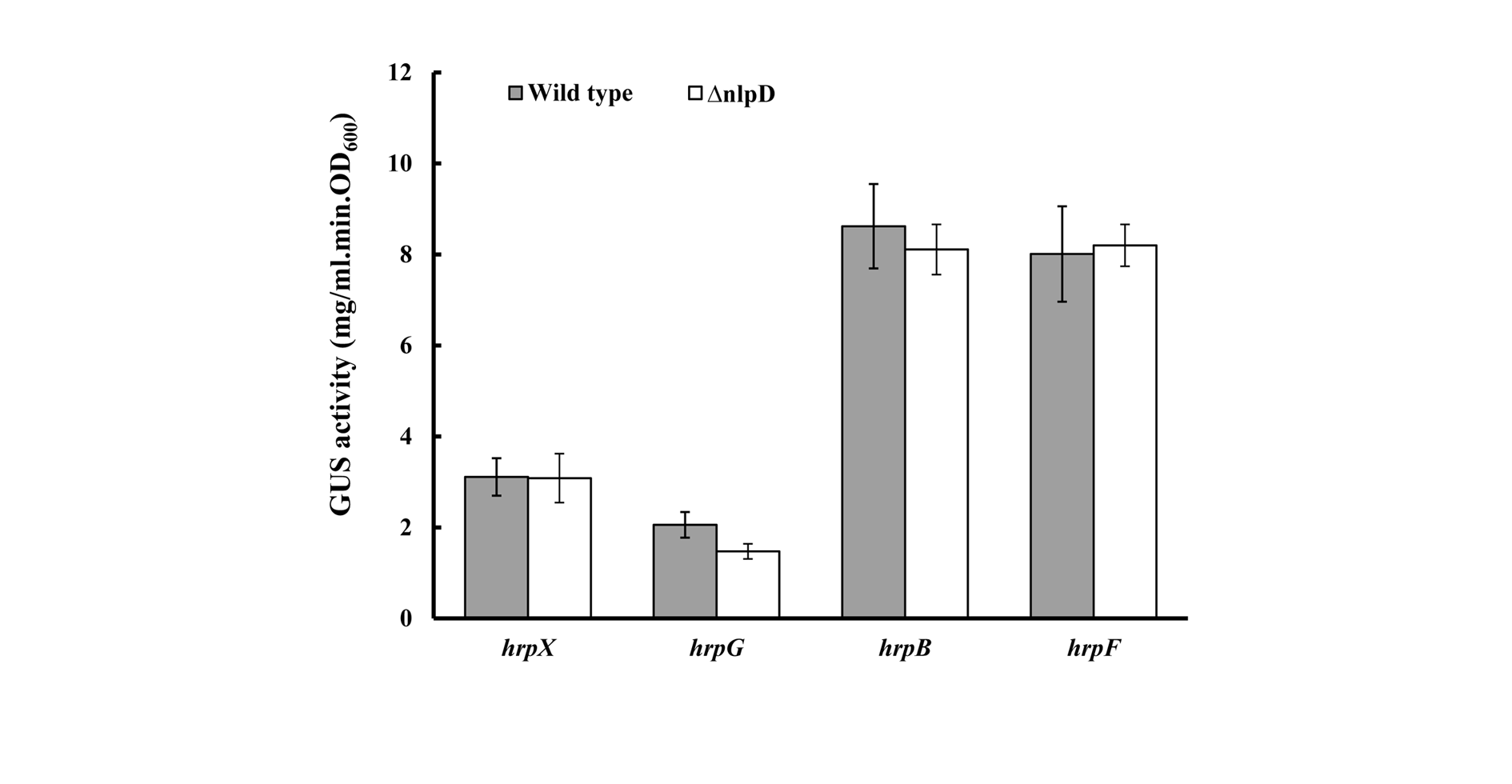

Supplement: Supplementary file 5 — Fig. S5 Mutation of nlpD does not affect the expression of hrp genes. The β‐glucuronidase (GUS) activities of hrpG, hrpX, hrpB and hrpF promoter‐gusA reporters in nlpD deletion (ΔnlpD) and wild‐type (Xcc 8004) backgrounds were determined. Xanthomonas campestris pv. campestris (Xcc) strains were cultured in the minimal medium XCM2 for 20 h, and the GUS activities were then determined by measurement of the absorbance at 415 nm using ρ‐nitrophenyl‐β‐d‐glucuronide as substrate. Data are the means ± standard deviations of triplicate measurements. The experiment was repeated three times, and similar results were obtained. [file MPP-19-1705-s005.tif]

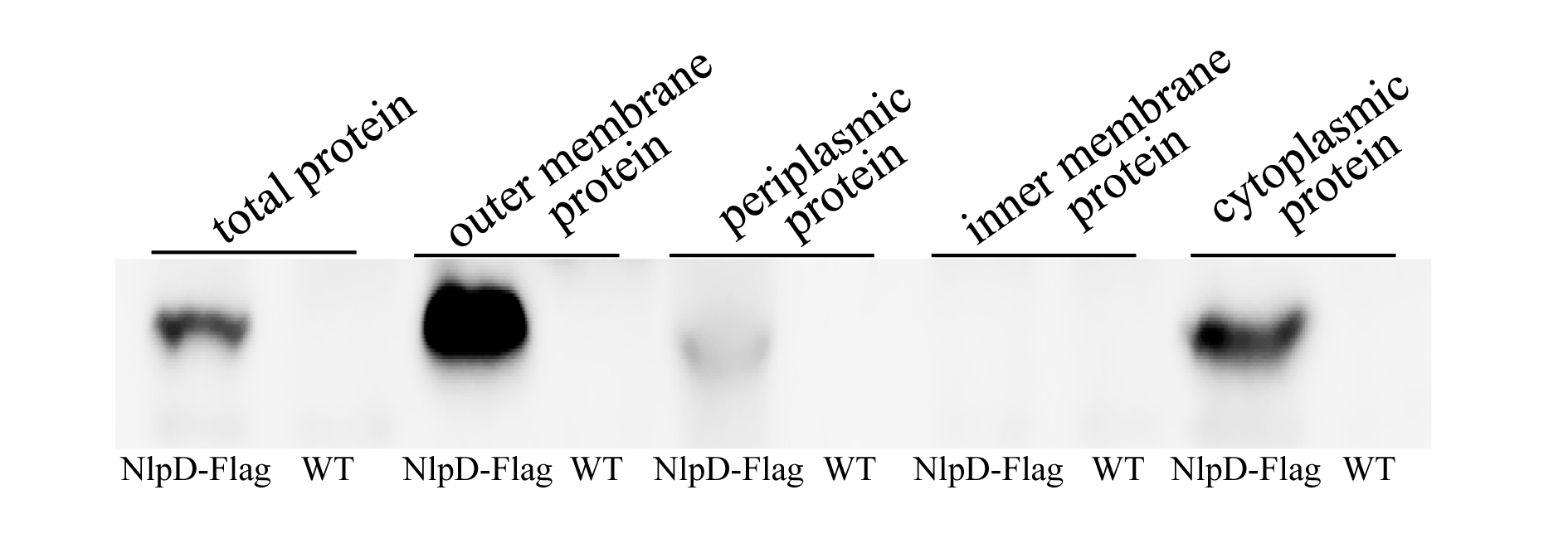

Supplement: Supplementary file 6 — Fig. S6 Subcellular localization of Xanthomonas campestris pv. campestris (Xcc) NlpD by Western blot analysis. The bacterial total and periplasmic proteins, as well as the outer and inner membrane proteins, were prepared from 100 mL of Xcc cells grown in NYG medium overnight. Thirty micrograms of each protein sample were separated by sodium dodecylsulfate‐polyacrylamide gel electrophoresis (SDS‐PAGE) and transferred to a polyvinylidene difluoride (PVDF) membrane. Anti‐Flag‐tag was used to detect the presence of the recombinant protein NlpD‐Flag. NlpD‐Flag, protein from recombinant strain 8004NlpD‐Flag; WT, protein from the wild‐type strain 8004. [file MPP-19-1705-s006.tif]

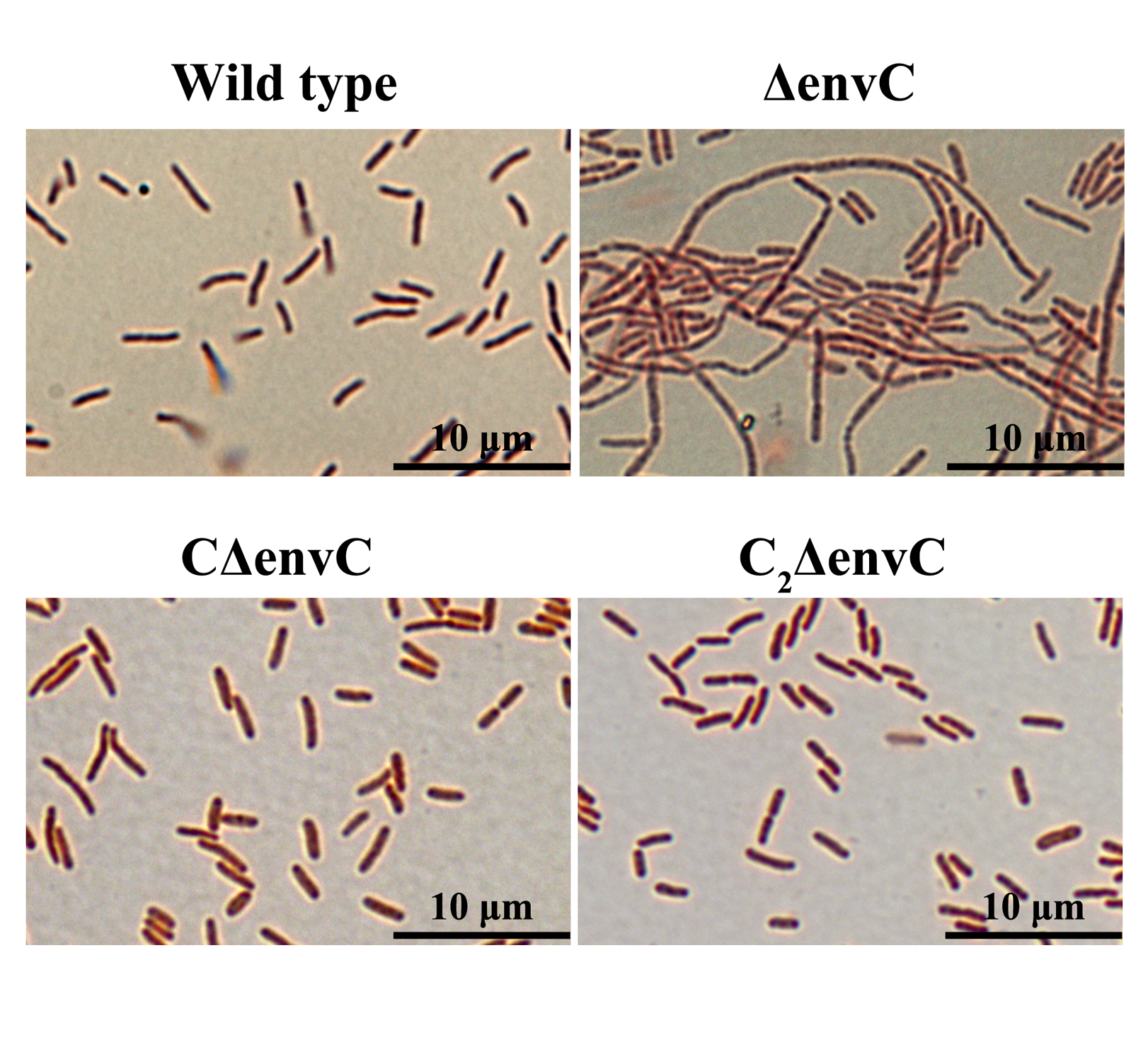

Supplement: Supplementary file 7 — Fig. S7 The mutant strain ΔenvC expressing in trans a recombinant gene encoding His6‐EnvCLN14 protein (strain C2ΔenvC) shows the wild‐type phenotype. Bacterial cells of Xcc strains cultured overnight in NYG medium were collected by centrifugation at 13 800 g for 30 s and resuspended in double‐distilled H2O. Five microlitres of bacterial resuspension were placed on a slide and dried at room temperature. After Gram staining, bacterial cells were observed by light microscopy. Representative micrographs are shown. [file MPP-19-1705-s007.tif]
