# Supplementary material for: Peptidoglycan hydrolysis mediated by the amidase AmiC and its LytM activator NlpD is critical for cell separation and virulence in the phytopathogen Xanthomonas campestris
Source: Mol Plant Pathol. 2018 Feb 1;19(7):1705–18. doi: 10.1111/mpp.12653 (PMC6638016; doi:10.1111/mpp.12653)
Supplement: Supplementary file 8 — Table S1 The predicted LytM proteins in Xanthomonas campestris pv. campestris (Xcc) and their homologues in Escherichia coli. [file MPP-19-1705-s008.doc]

| ***Xcc* strain 8004** | | | ***E. coli*** | | | **Amino acid Identity** |
| --- | --- | --- | --- | --- | --- | --- |
| **ID** | **Gene** | **Domain Structure** | **ID** | **Gene** | **Domain Structure** |  |
| ***XC_0022*** | ***envC*** | **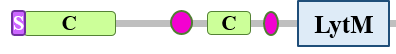** | ***b3613*** | ***envC*** | **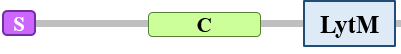** | **33%** |
| ***XC_2522*** | ***nlpD*** | **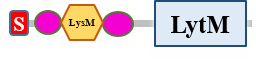** | ***b2742*** | ***nlpD*** | **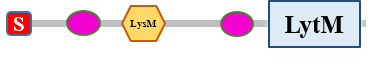** | **59%** |
|  |  |  | ***b2865*** | ***ygeR*** | **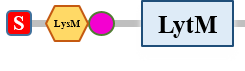** | **44%** |
| ***XC_3926*** | ***yebA*** | **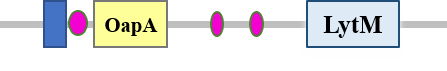** | ***b1856*** | ***yebA*** | **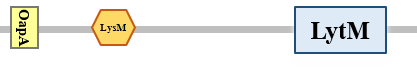** | **32%** |
| ***XC_0463*^a^** |  | **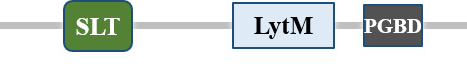** |  |  |  |  |
| ***XC_0921*** |  | **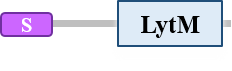** |  |  |  |  |
| ***XC_1250*** |  | **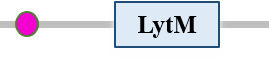** |  |  |  |  |
| ***XC_1354*** |  | **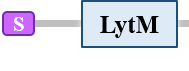** |  |  |  |  |
| ***XC_1857*** |  | **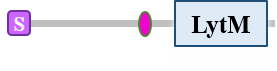** |  |  |  |  |
| ***XC_3502*** |  | **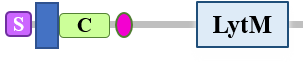** |  |  |  |  |
| ***XC_4282*** |  | **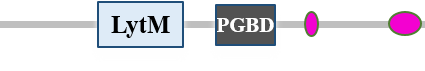** |  |  |  |  |

**Table S1. The predicted LytM proteins in *Xcc* and their homologues in *E. coli.***

^a^ *XC_0463* was annotated as a pseudogene. An insertion mutagenesis in *XC_0463* causes the premature termination of open reading frame (ORF). SMART analysis showed that the deduced protein of *XC_0463* contains 3 domains: SLT, LytM, and PGBD.


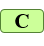
: Coiled coil region.


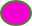
: Low complexity region.


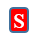
: This is a lipoprotein signal, as detected by the SPEPLip (<http://gpcr.biocomp.unibo.it/cgi/predictors/spep/pred_spepcgi.cgi>).


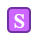
: This is a signal peptide, as detected by the SPEPLip (<http://gpcr.biocomp.unibo.it/cgi/predictors/spep/pred_spepcgi.cgi>).


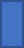
: This is a transmembrane helix region, as detected by the TMHMM v2.0 program.


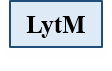
: This is a Pfam domain. Members of this family are zinc metallopeptidases with a range of specificities. The members of the peptidase M23 group belong to in this family.


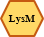
: This is a SMART LysM domain. The LysM (lysin motif) domain is about 40 residues long and found in a variety of enzymes involved in bacterial cell wall degradation.


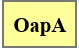
: This is a Pfam domain. This entry includes the *Haemophilus influenzae* opacity-associated protein. This protein is required for efficient nasopharyngeal mucosal colonization, and its expression is associated with a distinctive transparent colony phenotype. OapA is thought to be a secreted protein, and its expression exhibits high-frequency phase variation.


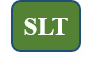
: This is a Pfam domain, which is found mainly in proteins from phages and type II, type III and type IV secretion systems. Bacterial lytic transglycosylases degrade murein via cleavage of the beta-1,4-glycosidic bond between N-acetylmuramic acid and N-acetylglucosamine, with the concomitant formation of a 1,6-anhydrobond in the muramic acid residue.


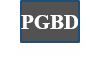
: This is a Pfam domain. This entry represents peptidoglycan binding domain (PGBD), as well as related domains that share the same structure. PGBD may have a general peptidoglycan binding function.
