# Supplementary material for: Peptidoglycan hydrolysis mediated by the amidase AmiC and its LytM activator NlpD is critical for cell separation and virulence in the phytopathogen Xanthomonas campestris
Source: Mol Plant Pathol. 2018 Feb 1;19(7):1705–18. doi: 10.1111/mpp.12653 (PMC6638016; doi:10.1111/mpp.12653)
Supplement: Supplementary file 9 — Table S2 Bacterial strains and plasmids used in this work. [file MPP-19-1705-s009.doc]

**Table S2. Bacterial strains and plasmids used in this work.**

| Strains or plasmids | Relevant characteristics | | Source | |
| --- | --- | --- | --- | --- |
| *Escherichia coli* |  | |  | |
| DH5*α* | *F^-^ Φ80lacZΔM15Δ*(*lacZYA-argF*) *U169 recA1 endA1 hsdR17*(*r_k_^-^*,*m_k_^+^*)*phoA supE44 thi-1 gyrA96 relA1 λ^-^* | Gibco BRL, Life Technologies | |  |
| BL21(DE3) | F^-^ *ompT hsdS_B_* (*r_B_^-^*, *m_B_^-^*) *gal dcm* (DE3) | | Novagen | |
| His_6_-NlpD_LN22_ | BL21(DE3) harboring pET-NlpD_LN22_, Kan^r^ | | This work | |
| His_6_-EnvC_LN14_ | BL21(DE3) harboring pET-EnvC_LN14_, Kan^r^ | | This work | |
| His_6_-AmiC1_LN32_ | BL21(DE3) harboring pET-AmiC1_LN32_, Kan^r^ | | This work | |
| His_6_-AmiC2_LN23_ | BL21(DE3) harboring pET-AmiC2_LN23_, Kan^r^ | | This work | |
|  |  | |  | |
| *Xcc* strains |  | |  | |
| *Xcc* 8004 | Wild type, Rif^r^ | |  | |
| NK0022 | *XC_0022* pK18mob integration mutant of 8004, the integration site lies in the 485^th^ bp downstream of the start codon of *XC_0022*, Rif^r^, Kan^r^ | | Our lab’s collection | |
| NK0463 | *XC_0463* pK18mob integration mutant of 8004, the integration site lies in the 510^th^ bp downstream of the start codon of *XC_0463*, Rif^r^, Kan^r^ | | Our lab’s collection | |
| NK0921 | *XC_0921* pK18mob integration mutant of 8004, the integration site lies in the 533^rd^ bp downstream of the start codon of *XC_0921*, Rif^r^ Kan^r^ | | Our lab’s collection | |
| 182E08 | *XC_1250* Tn5gusA5 insertion mutant of 8004, the insertion site lies in the 203^rd^ bp downstream of the start codon of *XC_1250*, Rif^r^, Kan^r^, Spc^r^, Gm^r^ | | Our lab’s collection | |
| NK1354 | *XC_1354* pK18mob integration mutant of 8004, the integration site lies in the 434^th^ bp downstream of the start codon of *XC_1354*, Rif^r^, Kan^r^ | | Our lab’s collection | |
| 032B12 | *XC_1857* Tn5gusA5 insertion mutant of 8004, the insertion site lies in the 661^st^ bp downstream of the start codon of *XC_1857*, Rif^r^, Kan^r^, Spc^r^, Gm^r^ | | Our lab’s collection | |
| NK2522 | *XC_2522* pK18mob integration mutant of 8004, the integration site lies in the 360^th^ bp downstream of the start codon of *XC_2522*, Rif^r^, Kan^r^ | | Our lab’s collection | |
| 209A07 | *XC_3502* Tn5gusA5 insertion mutant of 8004, the insertion site lies in the 111^st^ bp downstream of the start codon of *XC_3502*, Rif^r^, Kan^r^, Spc^r^, Gm^r^ | | Our lab’s collection | |
| NK3926 | *XC_3926* pK18mob integration mutant of 8004, the integration site lies in the 454^th^ bp downstream of the start codon of *XC_3926*, Rif^r^, Kan^r^ | | Our lab’s collection | |
| 024A02 | *XC_4282* Tn5gusA5 insertion mutant of 8004, the integration site lies in the 247^th^ bp downstream of the start codon of *XC_4282*, Rif^r^, Kan^r^, Spc^r^, Gm^r^ | | Our lab’s collection | |
| ∆hrcV | *hrcV* pK18mob integration mutant of 8004, the integration site lies in the 328^th^ bp downstream of the start codon of *hrcV*, Rif^r^, Kan^r^ | |  | |
| ∆envC | *envC* deletion mutant of 8004, deletion of the region from the 28^th^ to 1217^th^ bp downstream of the start codon of *envC*, Rif^r^ | | This work | |
| C∆envC | ∆envC harboring pJCenvC, Rif^r^, Tc^r^ | | This work | |
| C_2_∆envC | ∆envC harboring pJC_2_envC, Rif^r^, Tc^r^ | | This work | |
| ∆nlpD | *nlpD* deletion mutant of 8004, deletion of the region from the 33^rd^ to 780^th^ bp downstream of the start codon of *nlpD*, Rif^r^ | | This work | |
| C∆nlpD | ∆nlpD harboring pLCnlpD, Rif^r^, Tc^r^ | | This work | |
| amiC1::pK18 | *amiC1* pK18mob integration mutant of 8004, the integration site lies in the 360^th^ bp downstream of the start codon of *amiC1*, Rif^r^, Kan^r^ | | This work | |
| CamiC1::pK18 | ∆amiC1 harboring pJCamiC1, Rif^r^, Kan^r^, Tc^r^ | | This work | |
| ∆amiC2 | *amiC2* deletion mutant of 8004, deletion of the region from the 21^st^ to 1140^th^ bp downstream of the start codon of *amiC2*, Rif^r^ | | This work | |
| C∆amiC2 | ∆amiC2 harboring pJCamiC2, Rif^r^, Tc^r^ | | This work | |
| amiC1::pK18∆amiC2 | *amiC1* pK18mob integration mutant of ∆amiC2, Rif^r^, Kan^r^ | | This work | |
| amiC1::pK18∆amiC2/pJCamiC1 | ∆amiC1C2 harboring pJCamiC1, Rif^r^, Kan^r^, Tc^r^ | | This work | |
| amiC1::pK18∆amiC2/pJCamiC2 | ∆amiC1C2 harboring pJCamiC2, Rif^r^, Kan^r^, Tc^r^ | | This work | |
| 8004/pLGUShrpX | 8004 containing pLGUShrpX, Rif^r^, Tc^r^ | | This work | |
| 8004/pLGUShrpG | 8004 containing pLGUShrpG, Rif^r^, Tc^r^ | | This work | |
| 8004/pLGUShrpB | 8004 containing pLGUShrpB, Rif^r^, Tc^r^ | | This work | |
| 8004/pLGUShrpF | 8004 containing pLGUShrpF, Rif^r^, Tc^r^ | | This work | |
| ∆nlpD/pLGUShrpX | ∆nlpD containing pLGUShrpX, Rif^r^, Tc^r^ | | This work | |
| ∆nlpD/pLGUShrpG | ∆nlpD containing pLGUShrpG, Rif^r^, Tc^r^ | | This work | |
| ∆nlpD/pLGUShrpB | ∆nlpD containing pLGUShrpB, Rif^r^, Tc^r^ | | This work | |
| ∆nlpD/pLGUShrpF | ∆nlpD containing pLGUShrpF, Rif^r^, Tc^r^ | | This work | |
| 8004/pJXG3176 | 8004 containing pJXG3176, Rif^r^, Tc^r^ | | This work | |
| ∆nlpD/pJXG3176 | ∆nlpD containing pJXG3176, Rif^r^, Tc^r^ | | This work | |
| ∆hrcV/pJXG3176 | ∆hrcV containing pJXG3176, Rif^r^, Kan^r^, Tc^r^ | | This work | |
| ∆envC/pJXG3176 | ∆envC containing pJXG3176, Rif^r^, Tc^r^ | | This work | |
| ∆amiC2/pJXG3176 | ∆amiC2 containing pJXG3176, Rif^r^, Tc^r^ | | This work | |
| amiC1::pK18/pJXG3176 | amiC1::pK18 containing pJXG3176, Rif^r^, Kan^r^, Tc^r^ | | This work | |
| amiC1::pK18∆amiC2  /pJXG3176 | amiC1::pK18∆amiC2 containing pJXG3176, Rif^r^, Kan^r^, Tc^r^ | | This work | |
| 8004_NlpD-Flag_ | As *Xcc* 8004, the sequence of NlpD was replaced with *nlpD*-3×flag on chromosome, Rif^r^ | | This work | |
| Plasmids |  | |  | |
| pLAFR6 | Broad host range IncP cloning cosmid, Tc^r^ | |  | |
| pLAFRJ | Shuttle plasmid pLAFR3 derivate containing the multiple cloning sites of pUC19, Tc^r^ | |  | |
| pJXG | pLAFRJ containing 3×Flag, Tc^r^ | | Our lab’s collection | |
| pK18mob | Suicide plasmid in *X. campestris* pv. *campestris*, Kan^r^ | |  | |
| pK18mobsacB | Sucrose-sensitive suicide plasmid in *X. campestris* pv. *campestris*, Kan^r^ | |  | |
| pKD2522 | Fusion of the flanking fragments of the *XC_2522* were ligated into pK18mobsacB, Kan^r^ | | This work | |
| pK1816 | pK18mob containing a 378-bp internal fragment of *XC_1816*, Kan^r^ | | This work | |
| pKD0022 | Fusion of the flanking fragments of the *XC_0022* were ligated into pK18mobsacB, Kan^r^ | | This work | |
| pKD2472 | Fusion of the flanking fragments of the *XC_2472* were ligated into pK18mobsacB, Kan^r^ | | This work | |
| pLCnlpD | pLAFR6 containing complement fragment of *nlpD*, Tc^r^ | | This work | |
| pJCamiC1 | pLAFRJ containing complement fragment of *amiC1*, Tc^r^ | | This work | |
| pJCamiC2 | pLAFRJ containing complement fragment of *amiC2*, Tc^r^ | | This work | |
| pJCenvC | pLAFRJ containing complement fragment of *envC*, Tc^r^ | | This work | |
| pJC_2_envC | pLAFRJ containing DNA sequence encoding 6×His-tag fused with *envC* lacking the N-terminal 14 aa coding sequence, Tc^r^ | | This work | |
| pJXG3176 | pJXG containing ORF exclusive termination codon of *XC_3176*, Tc^r^ | | This work | |
| pLGUShrpX | pLAFR6 containing an *hrpX* promoter-*gus* fusion fragment, Tc^r^ | | Our lab’s collection | |
| pLGUShrpG | pLAFR6 containing an *hrpG* promoter-*gus* fusion fragment, Tc^r^ | | Our lab’s collection | |
| pLGUShrpB | pLAFR6 containing an *hrpB* promoter-*gus* fusion fragment, Tc^r^ | | Our lab’s collection | |
| pLGUShrpF | pLAFR6 containing an *hrpF* promoter-*gus* fusion fragment, Tc^r^ | | Our lab’s collection | |
| pET30a | Expression vector, N-terminal 6×His-tagged sequences, Kan^r^ | | Novagen | |
| pET-NlpD_LN22_ | pET30a containing the ORF lacking the N-terminal 22-aa signal peptide coding sequence of *nlpD*, Kan^r^ | | This work | |
| pET-EnvC_LN14_ | pET30a containing the ORF lacking the N-terminal 14-aa signal peptide coding sequence of *envC*, Kan^r^ | | This work | |
| pET-AmiC2_LN23_ | pET30a containing the ORF lacking the N-terminal 23-aa signal peptide coding sequence of *amiC2*, Kan^r^ | | This work | |
| pET-AmiC1_LN32_ | pET30a containing the ORF lacking the N-terminal 32-aa signal peptide coding sequence of *amiC1*, Kan^r^ | | This work | |

^a^ Gm^r^, Kan^r^, Rif^r^, Spc^r^ and Tc^r^ = Gentamycin-, Kanamycin-, Rifampicin-, Spectinomycin- and Tetracycline-resistant, respectively.

**Huynh, T.V., Dahlbeck, D. and Staskawicz, B.J.** (1989) Bacterial blight of soybean: regulation of a pathogen gene determining host cultivar specificity. *Science*. **245**, 1374-1377.

**Jiang, B.L., Liu, J., Chen, L.F., Ge, Y.Y., Hang, X.H., He, Y.Q., Tang, D.J., Lu, G.T. and Tang, J.L.** (2008) DsbB is required for the pathogenesis process of *Xanthomonas* *campestris* pv. *campestris*. *Mol. Plant-Microbe Interact.* **21**, 1036-1045.

**Jiang, W., Jiang, B.L., Xu, R.Q., Huang, J.D., Wei, H.Y., Jiang, G.F., Cen, W.J., Liu, J., Ge, Y.Y., Li, G.H., Su, L.L., Hang, X.H., Tang, D.J., Lu, G.T., Feng, J.X., He, Y.Q. and Tang, J.L.** (2009) Identification of six type III effector genes with the PIP box in *Xanthomonas* *campestris* pv. *campestris* and five of them contribute individually to full pathogenicity. *Mol. Plant-Microbe Interact.* **22**, 1401-1411.

**Qian, W., Jia, Y., Ren, S.X., He, Y.Q., Feng, J.X., Lu, L.F., Sun, Q., Ying, G., Tang, D.J., Tang, H., Wu, W., Hao, P., Wang, L., Jiang, B.L., Zeng, S., Gu, W.Y., Lu, G., Rong, L., Tian, Y., Yao, Z., Fu, G., Chen, B., Fang, R., Qiang, B., Chen, Z., Zhao, G.P., Tang, J.L. and He, C.** (2005) Comparative and functional genomic analyses of the pathogenicity of phytopathogen *Xanthomonas* *campestris* pv. *campestris*. *Genome Res*. **15**, 757-767.

**Schäfer, A., Tauch, A., Jäger, W., Kalinowski, J., Thierbach, G. and Pühler, A.** (1994) Small mobilizable multi-purpose cloning vectors derived from the *Escherichia coli* plasmids pK18 and pK19: selection of defined deletions in the chromosome of *Corynebacterium* *glutamicum*. *Gene*. **145**, 69-73.
