# Supplementary material for: Peptidoglycan hydrolysis mediated by the amidase AmiC and its LytM activator NlpD is critical for cell separation and virulence in the phytopathogen Xanthomonas campestris
Source: Mol Plant Pathol. 2018 Feb 1;19(7):1705–18. doi: 10.1111/mpp.12653 (PMC6638016; doi:10.1111/mpp.12653)
Supplement: Supplementary file 10 — Table S3 Quantification of the extracellular enzymes produced by Xanthomonas campestris pv. campestris (Xcc) strains. [file MPP-19-1705-s010.doc]

**Table S3. Quantification of the extracellular enzymes produced by *Xcc* strains.**

| Strains | Extracellular amylase (U) | Extracellular endoglucanase (U) | Extracellular protease (A_366_) |
| --- | --- | --- | --- |
| *Xcc* 8004 | 0.25 ± 0.01 A | 0.49 ± 0.11 A | 0.15 ± 0.00 A |
| ΔnlpD | 0.29 ± 0.09 A | 0.46 ± 0.05 A | 0.14 ± 0.01 A |
| CΔnlpD | 0.25 ± 0.04 A | 0.52 ± 0.07 A | 0.15 ± 0.01 A |
| ΔenvC | 0.25 ± 0.00 A | 0.57 ± 0.02 A | 0.17 ± 0.04 A |
| CΔenvC | 0.24 ± 0.01 A | 0.59 ± 0.06 A | 0.14 ± 0.01 A |
| ΔamiC2 | 0.21 ± 0.02 A | 0.54 ± 0.07 A | 0.15 ± 0.02 A |
| CΔamiC2 | 0.21 ± 0.03 A | 0.54 ± 0.07 A | 0.15 ± 0.01 A |
| amiC1::pK18 | 0.25 ± 0.04 A | 0.57 ± 0.07 A | 0.16 ± 0.02 A |
| CamiC1::pK18 | 0.23 ± 0.04 A | 0.47 ± 0.11 A | 0.16 ± 0.00 A |
| amiC1::pK18ΔamiC2 | 0.22 ± 0.02 A | 0.47 ± 0.04 A | 0.16 ± 0.01 A |
| amiC1::pK18ΔamiC2  /pJCamiC1 | 0.23 ± 0.06 A | 0.50 ± 0.06 A | 0.15 ± 0.01 A |
| amiC1::pK18ΔamiC2  /pJCamiC2 | 0.21 ± 0.02 A | 0.60 ± 0.08 A | 0.15 ± 0.02 A |

^*^ Data are the mean ± standard deviation of triplicate measurements; the letter A in each data column indicates no significant difference at *P* = 0.05. The experiment was repeated twice and similar results were obtained.
