# Supplementary material for: Peptidoglycan hydrolysis mediated by the amidase AmiC and its LytM activator NlpD is critical for cell separation and virulence in the phytopathogen Xanthomonas campestris
Source: Mol Plant Pathol. 2018 Feb 1;19(7):1705–18. doi: 10.1111/mpp.12653 (PMC6638016; doi:10.1111/mpp.12653)
Supplement: Supplementary file 11 — Table S4 The predicted N‐acetylmuramoyl‐l‐alanine amidases in Xanthomonas campestris pv. campestris (Xcc) and their homologues in Escherichia coli. [file MPP-19-1705-s011.doc]

**Table S4. The predicted N-acetylmuramoyl-L-alanine amidases in *Xcc* and their homologues in *E. coli.***

| ***Xcc* strain 8004** | | | ***E. coli*** | | | **Amino acid Identity** |
| --- | --- | --- | --- | --- | --- | --- |
| **ID** | **Gene** | **Domain Structure** | **ID** | **Gene** | **Domain Structure** |  |
| **XC1816** | ***amiC1*** | **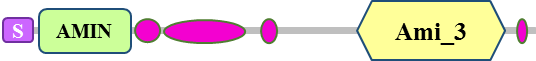** | **b2817** | ***amiC*** | **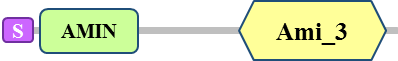** | **48%** |
| **XC2472** | ***amiC2*** | **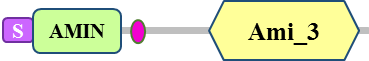** |  |  |  | **34%** |
|  |  |  | **b2435** | ***amiA*** | **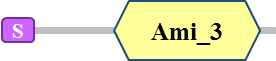** |  |
|  |  |  | **b4169** | ***amiB*** | **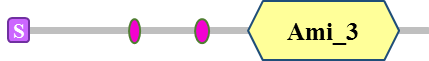** |  |
| **XC2695** | ***ampD*** | **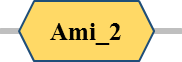** | **b0110** | ***ampD*** | **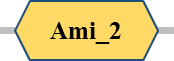** | **29%** |
| **XC3877** | ***amiD*** | **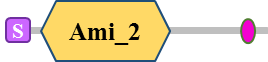** | **b0867** | ***amiD*** | **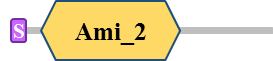** | **36%** |


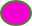
: Low complexity region.


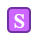
: This is a signal peptide, as detected by the SPEPLip (<http://gpcr.biocomp.unibo.it/cgi/predictors/spep/pred_spepcgi.cgi>).


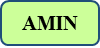
: This is a Pfam domain. This N-terminal domain of various bacterial protein families is crucial for the targetting of periplasmic or extracellular proteins to specific regions of the bacterial envelope. AMIN is derived from the N-terminal domain of AmiC, an N-acetylmuramoyl-l-alanine amidase of *Escherichia coli* which localises to the septal ring during division and plays a key role in the separation of daughter cells. The AMIN domain is present in several protein families besides amidases suggesting that AMIN may represent a general targetting determinant involved in the localisation of periplasmic protein complexes


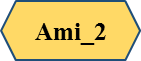
: This is a SMART Ami_2 domain. Proteins families containing this domain include zinc amidases that have N-acetylmuramoyl-L-alanine amidase activity (EC 3.5.1.28). This enzyme domain cleaves the amide bond between N-acetylmuramoyl and L-amino acids in bacterial cell walls (preferentially: D-lactyl-L-Ala). The structure is known for the Bacteriophage T7 structure and shows that two of the conserved histidines are zinc binding.


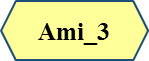
: This is a SMART Ami_3 domain. This enzyme domain cleaves the amide bond between N-acetylmuramoyl and L-amino acids in bacterial cell walls.
