# Supplementary material for: Peptidoglycan hydrolysis mediated by the amidase AmiC and its LytM activator NlpD is critical for cell separation and virulence in the phytopathogen Xanthomonas campestris
Source: Mol Plant Pathol. 2018 Feb 1;19(7):1705–18. doi: 10.1111/mpp.12653 (PMC6638016; doi:10.1111/mpp.12653)
Supplement: Supplementary file 12 — Table S5 Primers used in this work. [file MPP-19-1705-s012.doc]

**Table S5. Primers used in this work.**

| **Primer Name** | **Sequence** | **Product Length**  **(bp)** |
| --- | --- | --- |
| **For mutants construction** | | |
| D2522-LF(B) | AAAGGATCCCAAGAGGTTCTGGCGCCGGTC | 718 |
| D2522-LR(X) | AAATCTAGATGCGCCCTGCTCATCCGTTCG |  |
| D2522-RF(X) | GGGTCTAGACTGTATCTGCCCAAGAAGTGA | 722 |
| D2522-RR(H) | GGGAAGCTTAGATCATCGAACACACTGGCT |  |
| D0022-LF(E) | GGGGAATTC GCGGCTTGCGGTTCGGCTTG | 723 |
| D0022-LR(X) | GGGTCTAGA GCCGGCCAGCAGCGTGCAGG |  |
| D0022-RF(X) | GGGTCTAGA GCAGCGCCGCTGAGTGCATG | 698 |
| D0022-RR(H) | GGGAAGCTT CGTGCAGCCGGCGTGTCGTC |  |
| D2472-LF(B) | GGGGGATCC TCTTCATGATTTTGCCTTCCC | 696 |
| D2472-LR(X) | GGGTCTAGA AACACCTGCCGCCGCCTCAT |  |
| D2472-RF(X) | GGGTCTAGA GCATTTCCCGGTCGCGCCTG | 684 |
| D2472-RR(H) | GGGAAGCTT ACGCTGCGCTGGCTATGTGG |  |
| NK1816-F(B) | GGGGGATCC GTGATTGAATCCGGCTCCATGACC | 378 |
| NK1816-R(X) | GGGTCTAGA CGGCTTCAGCGCAGTGACTGGCG |  |
| D1816RR | GCCAGCGAACGCAGCCACTC |  |
| pKmob18Con | GCCGATTCATTAATGCAGCTGGCAC |  |
| C2522-F(B) | AAAGGATCC TTTCCGGTCCCGCCGGAAGTGA | 1359 |
| C2522-R(H) | GGGAAGCTT CAGCCCAAGGCCGTTACGATTC |  |
| C1816-F(E) | GGGGAATTC CCGGTGGCGAAGCCTGGCAT | 2046 |
| C1816-R(H) | GGGAAGCTT TCAGCGGCTGCCGCCGGCCA |  |
| C2472-F(E) | GGGGAATTC TGTGGTGGTCGCCGAGGTCG | 1526 |
| C2472-R(H) | GGGAAGCTT TCAGGCGCGACCGGGAAATG |  |
| C0022-F(E) | GGGGAATTC CGGGCATGCTCGCCAACCTGGATA | 1624 |
| C0022-R(H) | GGGAAGCTT TCAGCGGCGCTGCAGCCAGC |  |
| C0022-2F(E) | GGGGAATTC GCACCATCATCATCATCATCAGAGCCAGCGCGAGGCCGA | 1213 |
|  |  |  |
| **For subcellular localization** | | |
| Flag2522-LF(E) | GGGGAATTC GTGGAAGGCAACGAACGGAT | 816 |
| Flag2522-LR(B) | GGGGGATCC CTTCTTGGGCAGATACAGCA |  |
| Flag2522-RF(X) | GGGTCTAGA TGAAGCCGGGAGTCGGGAATG | 748 |
| Flag2522-RR(H) | GGGAAGCTT GGTGGCAGCGGAAGACCGTG |  |
| 3×Flag-F | GGGGGATCCGATTATAAAGATCATGACGGTGATTATAAAGATCATGACATCGACTACAAGGATGACGATGACAAGCTCGAGTGATCTAGAGGG |  |
| 3×Flag-R | CCCTCTAGATCACTCGAGCTTGTCATCGTCATCCTTGTAGTCGATGTCATGATCTTTATAATCACCGTCATGATCTTTATAATCGGATCCCCC |  |
|  |  |  |
| **For 5’ RACE** |  |  |
| 2522GSP1 | ACTTGCACCAGGCTTGCT |  |
| 2522GSP2 | GGGCGGATACAGCTTGAGCGTC |  |
| 2522GSP3 | ACAGCGTGTCGCCGCGCTGCAC |  |
| FP2522-F | ACAGCGGTGATGCTGGTAGTGGCGG |  |
| FP2522-R | CGCGCTGCACGGTCACGGTTGC |  |
|  |  |  |
| **For protein purification** | | |
| E2522-F(B) | AAAGGATCC GCGACAGTGGTGCGTTCGCCCA | 720 |
| E2522-R(E) | GGGGAATTC TCACTTCTTGGGCAGATACAGC |  |
| E1816-F(E) | GGGGAATTC GGCGAAATCAAGCAAGTGGA | 1614 |
| E1816-R(Xh) | AAACTCGAG TCAGCGGCTGCCGCCGGCCA |  |
| E0022-F(E) | GGGGAATTC CAGAGCCAGCGCGAGGCCGA | 1194 |
| E0022-R(Xh) | AAACTCGAG TCAGCGGCGCTGCAGCCAGC |  |
| E2472-F(E) | GGGGAATTC CAGCCGCAGCCGCTGCTACGCAGTT | 1110 |
| E2472-R(Xh) | GGGCTCGAG TCAGGCGCGACCGGGAAATG |  |
